# Active multiplexing for scalable generation and manipulation of photonic quantum states

**DOI:** 10.1186/s40580-026-00540-6

**Published:** 2026-02-25

**Authors:** Fumihiro Kaneda, Masahiro Yabuno

**Affiliations:** 1 Department of Physics, Graduate School of Science, Tohoku University, Sendai, Japan; 2 Advanced ICT Research Institute, National Institute of Information and Communications Technology, Kobe, Japan

**Keywords:** Photons, Multiplexing, Entanglement, Quantum information

## Abstract

Photons serve as essential carriers of quantum information in quantum computing, quantum networking, and quantum metrology. However, the lack of direct photon-photon interaction poses a fundamental barrier: the inherently probabilistic nature of nonlinear quantum operations. Active multiplexing has emerged as the critical strategy, transforming probabilistic nonlinear processes into deterministic building blocks for large-scale quantum systems. In this review, we explore the recent progress and potential architectures of photonic active multiplexing systems, ranging from the quasi-deterministic generation of single photons and complex entangled states to the realization of measurement-based quantum computing architectures. We also provide an overview of recent advancements in enabling hardware—including basic photon sources, detectors, switches, and their integration. By analyzing the state of the art and identifying key remaining challenges, we highlight how active multiplexing strategies serve as the cornerstone for the next generation of scalable photonic quantum information processing.

**Graphical abstract:**

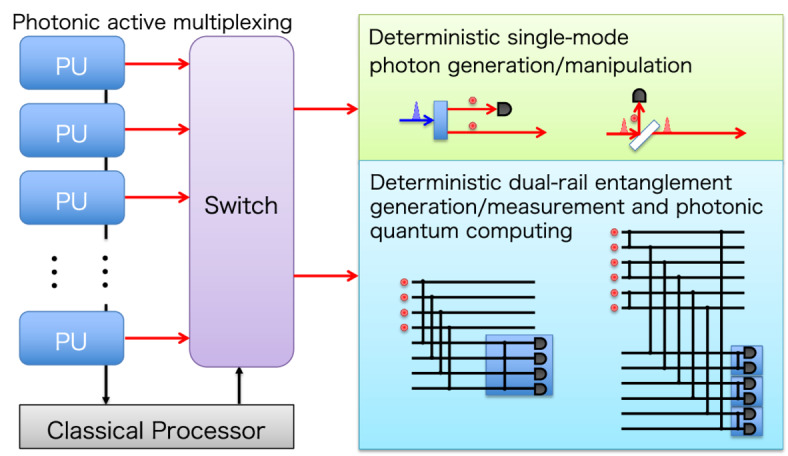

## Introduction

Quantum information science and technologies have been rapidly advancing, demonstrating the potential for efficient computing [1], secure communication [2], and precision measurement [3]. Photons, quantum particles of light, are excellent carriers of quantum information, such as quantum bits (qubits), thanks to their unique features of high traveling speed, stability at ambient temperatures, and mature techniques for their control, developed in the context of both classical and quantum optics. Thus, photons can be used not only for all-optical quantum information processing but also might be the only available option for connecting matter-based quantum devices [4, 5] placed remotely and their users via entanglement.

However, significant challenges remain for photons to be truly useful for quantum applications. First, deterministic sources of single-photon states and multi-photon entangled states have not yet been demonstrated (despite the recent rapid progress in the development of quantum light sources [6, 7], which will also be reviewed in this paper). Photon-pair generation via spontaneous parametric downconversion (SPDC) and spontaneous four-wave mixing (SFWM) has been widely used for the sources of two-photon entanglement [8] and heralded single photons [9]. Single-emitter sources based on semiconductor defects, quantum dots, and single atoms have the capability of deterministic generation of single photons. However, all of the sources lack either photon directionality (especially the capability of lossless coupling to single-mode optical fiber), indistinguishability, or generation probability. Second, photon-photon interactions are not realized in a direct manner, although they are essential for implementing two-photon quantum gates and photonic entanglement. The required optical nonlinearity for realizing a two-photon (controlled-phase) gate [10] is much higher than that observed in ordinary materials [11]. Cavity quantum electrodynamics (cavity QED) systems implemented by atomic systems [12, 13], as well as matter-based approaches utilizing quantum dots [14] and Rydberg atoms [15], have achieved such interactions, though often with restricted bandwidths and operational constraints. While high nonlinearity has been demonstrated in circuit QED with microwave photons [16], interfacing these systems with optical networks necessitates the development of quantum transduction technologies [17].

Another well-known approach to achieving high nonlinearity is to combine multi-photon interference with linear optics and the measurement of ancilla photons, as presented in the seminal work by Knill, Laflamme, and Milburn (KLM) [18]. This measurement-induced nonlinear operation is achieved only probabilistically, but detector signals herald successful events. Furthermore, the probabilistic nonlinear operations can be made deterministic through active multiplexing (MUX), where a sufficiently large number of probabilistic gates are operated in parallel or sequence to ensure the selection of a successful outcome. Since KLM’s initial discovery, MUX-based heralded schemes have been continuously improved and extended, serving as key techniques for the generation and manipulation of photonic quantum states and the construction of quantum information systems.

In this paper, we review MUX techniques that overcome the low optical nonlinearity and probabilistic nature of the generation and manipulation of photonic quantum states by *repeat-until-success* and/or *select-from-parallel* strategies. MUX is not just for resource-efficient implementations of photonic quantum systems, unlike ordinary optical communications and other classical information processing, but makes the probabilistic nonlinear process practically deterministic to scale up various photonic quantum information applications. While foundational MUX schemes have been successfully implemented and, in some cases, demonstrated supremacy over corresponding nonmultiplexed cases, many advanced architectures remain in the theoretical or early experimental phases. We review this exciting field by introducing the current state of the art and discuss remaining challenges. Moreover, we see the progress of hardware developments—such as fast optical switches, high-efficiency photon detectors, and high-quality single-photon sources—which have driven significant advancements in quantum protocols and application experiments. There are nice review articles on related topics, especially photonic quantum computing [1, 19–22] and MUX single-photon sources [6, 23]. This paper focuses on MUX as a highly promising avenue for scalability in the generation and manipulation of a wide range of photonic quantum states and aims to assist researchers by highlighting both the established advancements and the significant remaining challenges. By emphasizing these conceptual and theoretical frontiers, we intend to underscore the field’s high potential and the rich landscape for future research, contributing to the further acceleration of experimental advancement in the study of quantum information science with photons.

The rest of the paper is organized as follows: Sect. 2 briefly reviews the basic schemes, essential components, and necessity of MUX. Section 3 reviews various MUX schemes for the efficient generation of single-mode single photons and photon-number states, as well as photon-number manipulations. Section 4 discusses the generation and manipulation of dual-rail quantum operations, including entanglement generation via MUX, and furthermore, it presents photonic quantum computing schemes, which exploit MUX. Section 5 discusses the recent progress in developing key hardware components and explores their integration for stable, high-precision operations. Finally, Sect. 6 offers concluding remarks.

## MUX in photonic quantum systems

The inherently probabilistic nature of the required nonlinear quantum operations significantly restricts the scalability of photonic quantum applications. MUX technology enhances such probabilistic nonlinear operations, which are necessary because linear optics alone (e.g., beam splitters, mirrors, waveplates, phase shifters, and interferometers) cannot create the required photon-photon interactions, as they preserve the number of photons and adhere to linear transformations of the electromagnetic field.

**Fig. 1:**
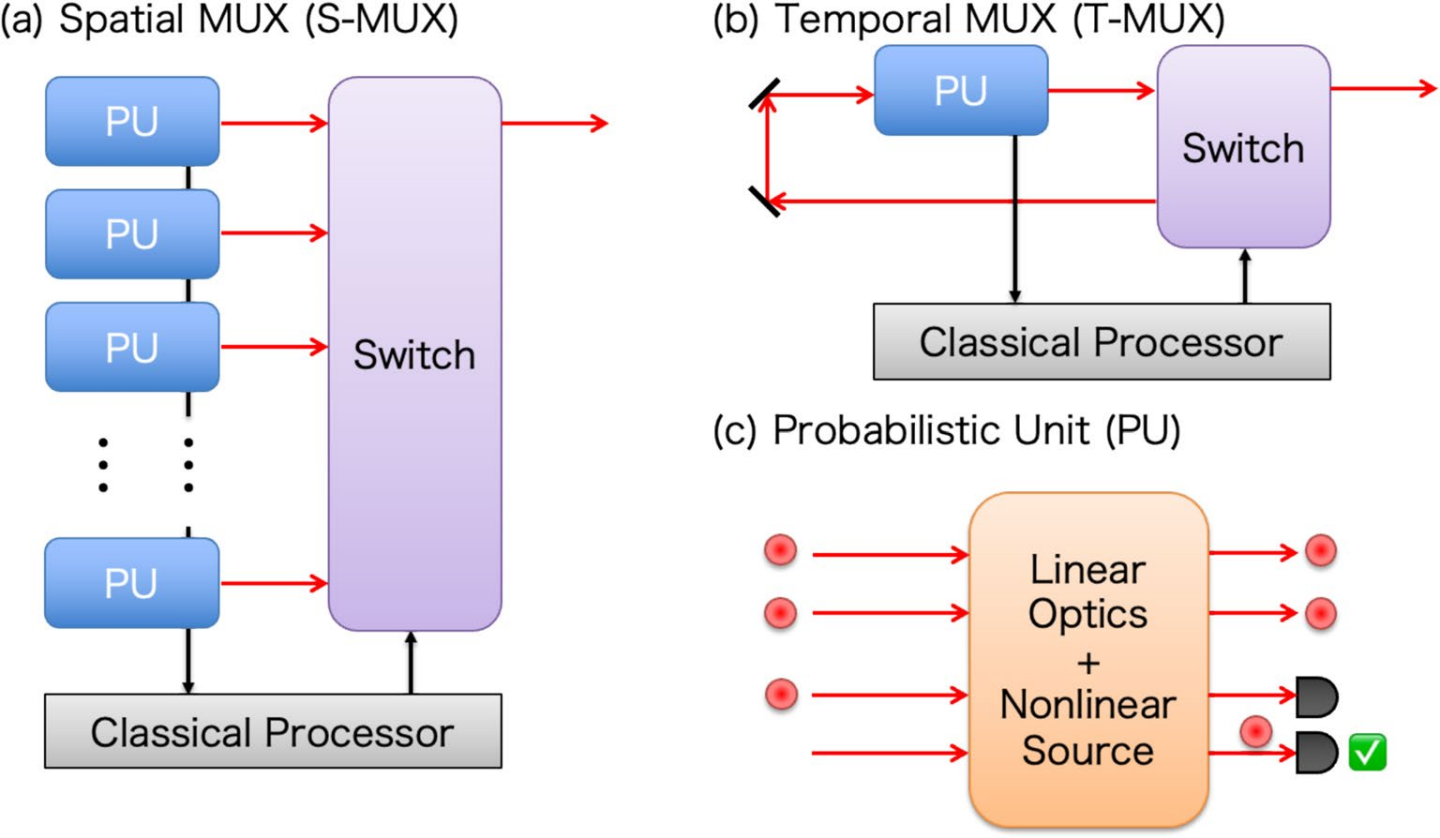
Conceptual diagram of photonic active multiplexing (MUX) schemes. **a** Spatial MUX (S-MUX) scheme. **b** Temporal MUX (T-MUX) scheme. **c** Probabilistic unit (PU)

Figure 1a and b show spatial MUX (S-MUX) and time MUX (T-MUX), respectively. We will see MUX in different optical degrees of freedom later, but S-MUX and T-MUX schemes have a longer research history [24, 25] and more advanced hardware development.

MUX takes a number of individually probabilistic units (PU) and combines them into a system that functions in a more deterministic manner. A PU is a quantum optical circuit designed to perform a specific operation—such as photon generation or a quantum gate—that succeeds only probabilistically. Physically, a PU typically comprises nonlinear optical sources (e.g., SPDC or SFWM), linear optical circuits, and photon detectors, as shown in Fig. 1c. While the operation itself is probabilistic, the key feature of a PU is that its success is *heralded*: the successful generation of a desired output state is signaled by specific click patterns from the detectors. The underlying mechanism enabling this heralding relies on preparing more photons and optical modes than are required for the final output state. The input state, involving these extra ancillary photons, undergoes a linear transformation within the PU. Specific detectors in the ancillary modes register photons, which are correlated (i.e., entangled) with the desired output state of the remaining unmeasured photons. Consequently, the successful operation of the PU is heralded without direct measurement of the output state, enabling its use in subsequent operations.

The MUX schemes utilize heralding signals from multiple PUs to achieve a higher success probability than in the single-PU case. In S-MUX, multiple PUs are prepared and operated in parallel. When at least one PU is heralded, an output quantum state from a heralded PU is adaptively routed to the multiplexed output port via an optical switch activated by a classical processor upon reception of the successful click patterns of detectors. When the number of PUs, i.e., the number of multiplexed modes *M*, is sufficiently high relative to the probability of heralding $p$, ($pM\gg 1$), a quasi-deterministic nonlinear quantum operation is enabled by MUX in *select-from-parallel* manners. In general, S-MUX schemes need more PUs and switches than non-MUX cases. Conversely, T-MUX can be operated with a single PU in an optical loop, which is repeated until a successful operation is heralded. After a heralded cycle, an output quantum state from the PU is released from the loop by optical switches. This *repeat-until-success* strategy is more resource-efficient than the S-MUX strategy, although its operational rate is reduced by the factor of multiplexed temporal modes *M* due to the sequential operation of a single PU. However, this reduced repetition rate is generally outweighed by the gain in the effective probability as the number of required nonlinear photon generation and manipulation increases. Note that the function of optical switches in MUX is not only the routing but also the linear transformation of heralded quantum states based on heralding signals. This essential role of feedforward operations for achieving quasi-deterministic nonlinear processes contrasts with matter-based quantum information platforms [1], where feedforward is mainly needed for error correction after deterministic quantum gates.

PUs can be categorized based on the complexity of the target states: those operating on single optical modes (e.g., generating single photons and Fock states) and those manipulating multi-mode entangled states (specifically, dual-rail encoded qubits). The efficient realization of the latter relies heavily on the deterministic supply of the former. Therefore, the following sections are organized to reflect this hierarchy: Sect. 3 reviews MUX techniques for single-mode resource generation, which serves as the foundation for Sect. 4, where we discuss MUX strategies for dual-rail entanglement and scalable quantum computing architectures.

We note that a PU can be constructed both locally in a single laboratory and across remote locations. A representative remote quantum operation is quantum teleportation [26–28], where one party (Alice) performs linear optical operations and photon detection to determine the feedforward signal, while the other party (Bob), located remotely, performs a single-qubit operation on his own photons to receive Alice’s qubit. Crucially, the success probability of quantum teleportation can be enhanced by the MUX of dual-rail entanglement generation and measurement techniques, as will be discussed in Sect. 4.1.

## MUX of single-mode photon-number operations

A key feature of the MUX technique is its ability to overcome the probabilistic generation of photonic quantum states in a single optical mode. Such techniques are generalized to photon-number manipulation. Here, we will explore various MUX techniques for generating (multiple) single-photon states, photon-number states, and photon-number entanglement, as well as photon addition and subtraction operations. These methods serve as fundamental techniques for the next section on the generation and manipulation of dual-rail multi-mode quantum states.

### MUX of heralded single-photon sources

**Fig. 2:**
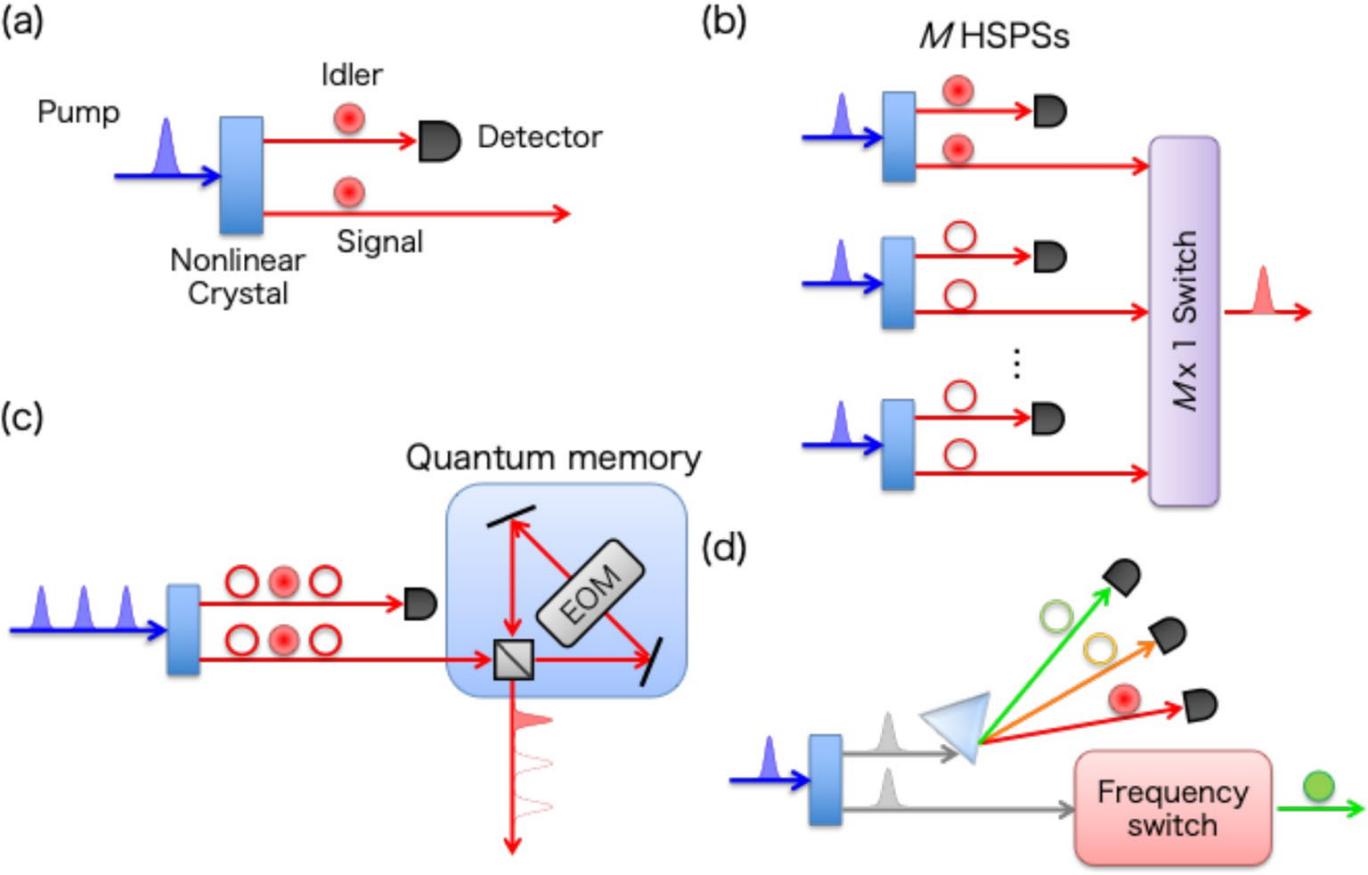
Conceptual diagram of MUX of heralded single-photon sources (HSPS). **a** HSPS serves as a PU, consisting of a nonlinear and probabilistic photon-pair source and a single-photon detector. **b** S-MUX HSPS. **c** T-MUX HSPS **d** F-MUX HSPS. MUX schemes in other optical degrees of freedom (e.g., polarization and orbital angular momentum) can also be realized

Single-photon sources are an essential resource of quantum information carriers for various applications. Here we see a heralded single-photon source (HSPS) as a PU, to which MUX techniques are applied straightforwardly. A conceptual diagram of HSPS is shown in Fig. 2a. In HSPS, a signal photon and its twin (idler) photon are produced via SPDC and SFWM. Signal and idler photons are produced as a two-mode squeezed vacuum (TMSV) state, where the photon numbers of signal and ancilla photons are identical: $|\mathrm{TMSV}\rangle ={\sum }_{n}\sqrt{{p}_{n}}|n\rangle |n\rangle$, where $|n\rangle$ is a *n*-photon Fock state and ${p}_{n}={\mu }^{n}/{\left(1+\mu \right)}^{n+1}$ and $\mu$ is a mean photon number of TMSV state. The idler mode serves as an ancilla mode of PU, directly subjected to the measurement of its photon number, which is the same as that of the signal mode. Therefore, single-photon detection in the idler mode heralds the presence of a signal photon. The HSPS was first demonstrated in the 1980s [9, 29], and since then, the technology has been widely applied to various experiments. Photon collection efficiency into single-mode optical fiber and source-to-source spectral indistinguishability, which are essential for performing two-photon linear-optical quantum gates, are reaching near unity [30, 31] (the technological improvements of HSPSs will be discussed in Sect. 5). However, from a single HSPS the theoretical maximum probability of emitting a heralded single-photon state is limited by 25% due to the photon statistics of the TMSV state (i.e., ${p}_{n}\le 0.25$ and maximized for $\mu =1$). Crucially, practical HSPSs are constrained by a fundamental trade-off between purity and efficiency: maintaining high single-photon fidelity (i.e., suppressing multi-photon contamination) necessitates keeping the mean photon number $\mu$ low, which inherently limits the heralding probability ${p}_{1}$. Furthermore, a practical HSPS typically uses a threshold detector, which binarily distinguishes $|0\rangle$ and $|n>0\rangle$, necessitating small $\mu$ and postselective measurements for mitigating photon-number uncertainty. A photon-number-resolving detector (PNRD, which will be discussed in Sect. 5) allows for a more precise measurement of the TMSV state, although any sub-unity detection efficiency inevitably results in residual photon-number uncertainty.　This fundamental purity-efficiency constraint is the primary bottleneck that MUX schemes aim to overcome, as it strictly limits the scalability in multi-photon generation.

MUX of HSPSs can efficiently overcome their probabilistic nature, enabling a quasi-deterministic single-photon generation. In MUX-HSPS, multiple HSPSs (which can be implemented in one or multiple nonlinear sources, as shown in Fig. 2b–d) are pumped, and a signal photon from a heralded source is adaptively transformed to a predetermined multiplexed mode by optical switches. In this case, the single-photon generation probability is no longer constrained by the 25% limit of a single nonlinear source, reaching as high as a multiplexed heralding probability ${P}_{H1}$, which is calculated as the probability that one or more HSPSs are heralded:

$${P}_{H1}=1-{\left(1-{p}_{1}\right)}^{M}, (1)$$

where *M* is the number of multiplexed modes. In Eq. (1), lossless detectors and ${p}_{1}\ll 1$ are assumed for simplicity: theoretical analysis of MUX-HSPS with imperfect components is given in [6, 32–34]. For a sufficiently large number of *M*, one can achieve the quasi-deterministic generation of heralding signals (${P}_{H1}\to 1$) and thereby heralded single photons; for example, one needs *M* = 17 and ${p}_{1}=25\%$(with an ideal PNRD) to achieve *P*_*H*1_ > 99%. In practice, due to optical loss (in both HSPSs and optical switches), the generation probability of the heralded single photon *P*_*G*1_ at the output of the optical switch is less than ${P}_{H1}$ [32–34]. Nonetheless, as shown below, multiple MUX experiments have already demonstrated superior performance over the non-multiplexed cases.

S-MUX of HSPS was proposed by Migdall and co-workers [24] in 2002. As shown in Fig. 2b, this scheme uses *M* identical HSPSs excited simultaneously, and heralded photons are then routed into a predetermined single optical path through an $M\times 1$ switch. S-MUX-HSPS has been experimentally demonstrated with bulk optics [35–37], fiber optics [38–40], and chip-scale platforms [41] (experiments in [36] and [39] additionally demonstrated MUX of two polarization modes and four temporal modes, respectively). The largest number of demonstrated multiplexed spatial modes is *M* = 6 in [37]. However, although these experiments observed an enhancement in the heralding probability *P*_*H*1_, the single-photon generation probability *P*_*G*1_ did not reach the 25% limit of a single nonlinear source due to significant losses in optical components. For both bulk and integrated optics platforms, a typical $M\times 1$ optical switch is implemented using a binary-tree architecture, with (*M* – 1) $2\times 2$ optical switches based on acoustic-optic modulators (AOM) and electro-optic modulators (EOM) [42]. Thus, heralded single photons typically pass through ~${\mathrm{log}}_{2}M$ switches. Integrated optics with reduced waveguide loss (which will be discussed in Sect. 5) will be a suitable platform for implementing a large number of HSPS and switches for S-MUX-HSPS. The optimization of switch networks has also been studied for minimizing switch assembly [23]. A low-loss dump-the-pump S-MUX scheme [43] can also implement S-MUX-HSPS with reduced loss by switching pump laser pulses of sequentially aligned HSPSs. The scheme can avoid a lossy direct switching process for heralded single photons, despite the need for low-latency control of pump laser pulses during the interval between subsequent pumping of HSPS.

T-MUX-HSPS was also first proposed and experimentally demonstrated in 2002 by Pittman and co-workers [25]. This scheme requires only one HSPS and one optical-loop quantum memory, and thus, is more resource-efficient than the S-MUX-HSPS. As shown in Fig. 2c, an HSPS is repeatedly pumped by *M* optical pulses with a period *τ*. When one (or more) single photons are heralded by the *M* optical pulses, one of the heralded single photons is loaded in the optical-loop quantum memory, where a single photon can be delayed for arbitrary integer multiples of *τ*. After pumping the HSPS *M* times, a heralded stored photon is released at a predetermined time; for example, in *M*-mode T-MUX-HSPS, a single photon heralded at *m*-th temporal mode ($m\le M$) is stored in the loop for $M-m$ cycles. This operation multiplexes HSPSs distributed across *M* temporal modes into a single mode. Note that, as shown in Fig. 2c, the T-MUX-HSPS allows the HSPS to be positioned outside the optical loop—in contrast to the general T-MUX configuration shown in Fig. 1b—because the HSPS serves as a PU for a vacuum input state. This configuration mitigates losses incurred by multiple passes through the nonlinear crystal and other optics within the HSPS. The simplicity of HSPS allows the implementation of T-MUX using a digital adjustable delay line [39, 41] without photon recycling. Despite the advantage of the resource-efficient implementation, T-MUX schemes need to be operated with a reduced operational rate by a factor of *M* compared to the HSPS. However, this factor-*M* decrease in the repetition rate is generally outweighed by the gain in the effective generation probability of generating multiple single photons. Since the *N*-single-photon generation rate scales linearly with the repetition rate but as the *N*-th power of *P*_*G*1_, the substantial boost in *P*_*G*1_ makes MUX sources far more efficient at producing multiple single photons.

Since the first experimental demonstration of enhanced *P*_*H*1_ in T-MUX-HSPS by Pittman and co-workers [25], this method has been theoretically analyzed and extended [32, 44, 45]. By low-loss HSPS (30%) and storage loop (3% per cycle) with a bulk EOM, the T-MUX source demonstrated for up to *M* = 30 with *P*_*H*1_ > 98% and *P*_*G*1_ = 38% [34]. The bulk-optics scheme was further improved (with 9% loss in an HSPS and 1.2% loss per cycle in the storage loop), achieving *M* = 40, *P*_*H*1_ > 98%, and *P*_*G*1_ = 67% [33]. Currently, those bulk-optic T-MUX-HSPSs are the only ones truly overcoming the ideal non-MUX counterpart (i.e., ${p}_{1}\le 25\%$ for TMSV states). The source needs a high mean photon number (*μ* = 0.18) of HSPS that is accompanied by a high likelihood of multi-photon contamination (the corresponding second-order correlation function *g*^(2)^ = 0.27). The photon-number uncertainty can be largely mitigated by employing a high-efficiency PNRD technology to improve the precision of photon-number measurement, as will be discussed in Sect. 5. The T-MUX HSPS has also been demonstrated with fiber-based loop [46], adjustable fiber-based delay line [39, 41], and concatenated optical loops [47].

MUX of HSPSs has also been demonstrated by using frequency [48, 49] and orbital angular momentum (OAM) [50] degrees of freedom. These schemes are unique in that a MUX system can be implemented using only one HSPS and one multi-mode switch. As shown in Fig. 2d, in frequency MUX (F-MUX), a nonlinear source produces spectrally correlated signal and idler photons, and the presence of a signal photon and its spectral mode is heralded by spectrally resolved detection of the idler mode. The spectral state of heralded signal photons is then adaptively switched via nonlinear wavelength conversion. Remarkably, multi-mode nonlinear wavelength conversion of single photons has been demonstrated by EOM with high modulation frequency and depth [49] and Bragg-scattering four-wave mixing (BSFWM) [48] with a single device. This approach offers a fundamental advantage over S-MUX (where the number of $2\times 2$ switches scales with *M*) and T-MUX (where the operational rate scales inversely with *M*). The multi-mode conversion is performed in a single device, resulting in a fixed insertion loss regardless of the number of multiplexed modes *M*. A frequency MUX-HSPS has been demonstrated for up to *M* = 3 with the enhancement of *P*_*H*1_. However, their multiplexed modes and large insertion loss (5 dB in EOM and 1.3 dB in BSFWM) need to be improved to overcome the single-HSPS limit. A proof-of-concept experiment of MUX-HSPS in orbital angular momentum (OAM) for up to *M* = 3 has also been demonstrated by using a spatial light modulator as a multi-mode spatial mode converter, but its switching efficiency (~ 30%) and bandwidth (~ 100 Hz) need to be largely improved to perform adaptive switching of OAM modes.

MUX of multiple degrees of freedom is advanced in that a large number of *M* can be obtained as a product of multiplexed modes in each degree of freedom, by exploiting the features of different MUX schemes. MUX of *M* = 4 hybrid modes with 2 polarization modes and 2 spatial modes has been efficiently implemented by using one polarization switch and one spatial switch [36]. *M* = 8 hybrid modes with 4 temporal modes and 2 spatial modes have been demonstrated by using an adjustable delay line with 2 input and 2 output ports [39].

**Fig. 3:**
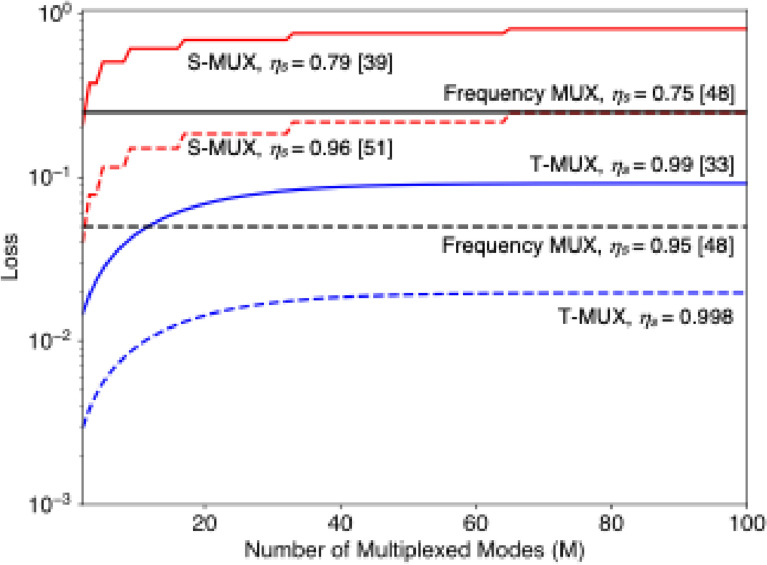
Switching loss versus multiplexed modes *M* for the S-MUX, T-MUX, and F-MUX strategies. The total loss (1 − transmittance) is plotted to emphasize small performance differences in the high-efficiency regime. The values of the single-switch transmittance *μ*_*s*_ are based on reported performances of demonstrated MUX-HSPSs (solid) and potential near-term improvements (dashed). For the T-MUX schemes, the heralding probability per pulse is set to *p* = 0.08, which is derived from a mean photon number *μ* = 0.1 for the TMSV state and a detector efficiency of *μ*_*d*_ = 0.95. The potential improvement of the T-MUX scheme is estimated from off-the-shelf, high-quality anti-reflection coatings on EO crystals

**Fig. 4:**
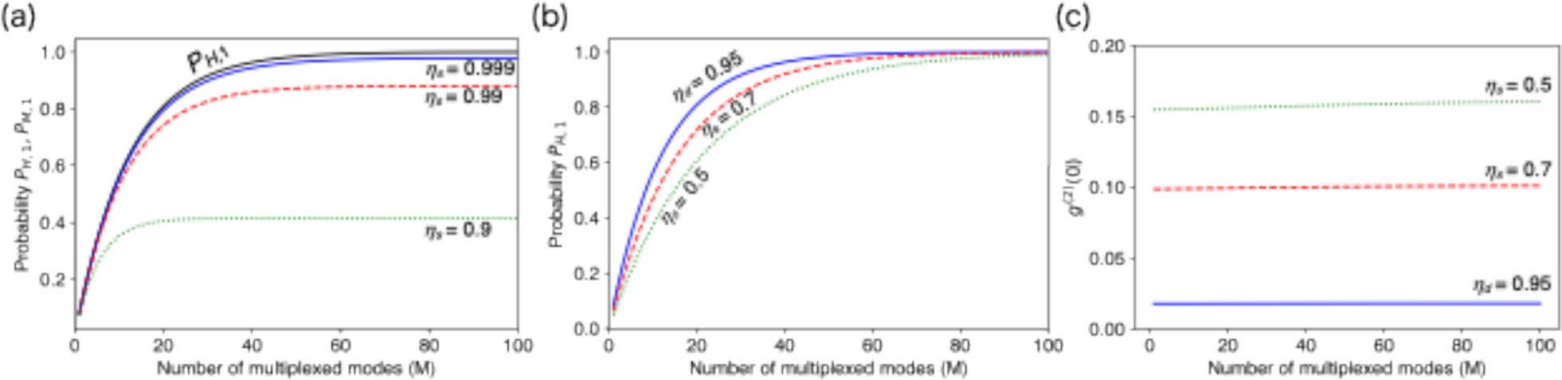
Performance of loop-based T-MUX-HSPS versus multiplexed modes *M*. **a** Single-photon generation probability *P*_*M*1_. **b** Heralding probability *P*_*H*1_. **c** Second-order autocorrelation function g^(2)^(0). *P*_*M,1*_ is highly dependent on the efficiency of the loop (*μ*_*s*_), while higher detector efficiency (*μ*_*d*_) is required for the saturation of *P*_*H,*1_ (and thereby *P*_*M,*1_) with a smaller number of *M* and lower *g*^(2)^(0). For those plots, a true PNRD and lossless optics are assumed. *μ*_*d*_ = 0.95 for **a** and *μ*_*d*_ = 0.99 for **b**, **c**. We set a mean photon number *μ* = 0.1 of the TMSV

The performance of MUX-HSPS can be evaluated by considering the HSPS units and the switching fabric separately. The choice of a MUX strategy depends heavily on the switching performance when scaling to a large number of modes *M* (experimental performances of previous works are summarized in [6]). Figure 3 illustrates the loss versus *M* with the single-switch transmittance $${\upeta }_{s}$$ for different MUX strategies. Here, we plot the total loss rather than the transmittance to clearly visualize the small but critical performance differences, especially in the high-efficiency regime. We compare loss scaling using $${\eta }_{s}$$ reported for demonstrated MUX-HSPSs, alongside potential near-term improvements. The total loss of a binary-tree-type *M*-mode switch in the S-MUX is described as $$1-{\eta }_{s}^{\lceil{\mathrm{log}}_{2}M\rceil}$$. For our analysis, we assume $${\eta }_{s}$$ values from waveguide-integrated EOMs [39, 51], which are essential for scalable S-MUX implementation. The T-MUX scheme utilizes a loop-based memory where the number of storage cycles depends on the heralding probability per pulse *p*. The expected loss is given by the weighted average: $$1-\sum_{m=1}^{M}{p{(1-p)}^{M-m}\eta }_{s}^{M-m}/ \sum_{m=1}^{M}p{(1-p)}^{M-m}$$. In Fig. 3, we set *p* = 0.08, which is derived from a mean photon number of *μ* = 0.1 for the TMSV state and a detector efficiency of *μ*_*d*_ = 0.95. These parameters are consistently applied to the plots in Fig. 4. The detailed calculation of $p$ is provided in Refs. [32–34]. For F-MUX using BSFWM, a single switch can ideally support *M* modes, making the total loss $$1-{\eta }_{s}$$ independent of *M*. Currently, the T-MUX scheme outperforms the other strategies due to the high efficiency of existing loop-based configurations. However, the superior scaling with *M* for the S-MUX and F-MUX strategies becomes more favorable if the loss per switch is further reduced to levels comparable to those of T-MUX components.

Figure 4 illustrates the dependence of hardware components in the T-MUX-HSPS as an example (the theory of the T-MUX-HSPS is described in [32–34]). As depicted in Fig. 3, the single-photon generation probability *P*_*G*1_ is highly reliant on the switch efficiency in the loop-based memory ${\eta }_{s}$, where a single photon is transmitted repeatedly in MUX operations. Meanwhile, the detector efficiency ${\eta }_{d}$, which is in an HSPS and is responsible for efficient and precise heralding of single-photon generation, is critical to achieve the saturation of *P*_*H*1_ and *P*_*G*1_ with fewer *M* and lower *g*^(2)^(0). The dependency on ${\eta }_{d}$, is similarly applied to the other types of MUX-HSPS. These clearly underscore the necessity of high-performance hardware components even for single-photon generation, and integrating those into a MUX system will enable a photonic device with unprecedented performance.

We note that single-photon sources based on semiconductor quantum dots [7] have also made rapid progress in recent years, demonstrating advanced quantum experiments [52–55]. Such a device, serving as an artificial atom, can be deterministically excited, typically by optical means [56–60] and decayed via single-photon emission. The high excitation-to-collection efficiency (> 70%), the second-order correlation function *g*^(2)^ (0) < 0.02, and indistinguishability > 98% between sequentially emitted photons from a single source have been simultaneously achieved by the current state of the art [57, 59, 60]. However, the simultaneous achievement of indistinguishability of photons from different sources remains an ongoing challenge [61], which hinders the use of multiple sources and limits the true scalability. One of the pathways to avoid the issue is to use a single source and perform serial-to-parallel conversion, the reverse operation of S-MUX, in which sequentially produced single photons are distributed to different optical paths and simultaneously input into optical circuits [52, 62]. This approach highlights that even for deterministic sources, switching architectures utilized in MUX remain crucial for providing the mode transformation necessary for scalable multi-photon experiments.

### MUX of multi-photon generation

Simultaneous generation of multiple single photons is an essential technology to scale up quantum information applications and can be achieved by a straightforward extension of MUX-HSPS. By employing an array of *N* independent sets of MUX-HSPSs, the simultaneous generation probability of single photons from each MUX source is ${P}_{GN}={P}_{G1}^{N}$, which offers significant enhancement over the ideal non-multiplexed case (${p}_{1}^{N}={0.25}^{N}$). The scheme using relative T-MUX (R-T-MUX) of *N* HSPSs [63–68] can be even more efficient than simply operating multiple T-MUX-HSPSs: For heralded multiple single photon generation, each T-MUX-HSPS does not need to be operated with a certain period, but can immediately emit heralded single photons after all T-MUX-HSPSs are heralded. This R-T-MUX procedure helps boost the operational rate of *N* single-photon generation. R-T-MUX of *N* = 2 photons [63–66] has been experimentally demonstrated, and the experiment in [63] demonstrated the multiplexing of *M* = 40 temporal modes of two HSPSs, achieving a 30-fold increase in simultaneous two-photon generation. The same experimental setup has also successfully enhanced the secure key rate in measurement-device-independent quantum key distribution (MDI-QKD) [69], where secret key generation relies on the projection of the two single photons into an entangled state.

**Fig. 5:**
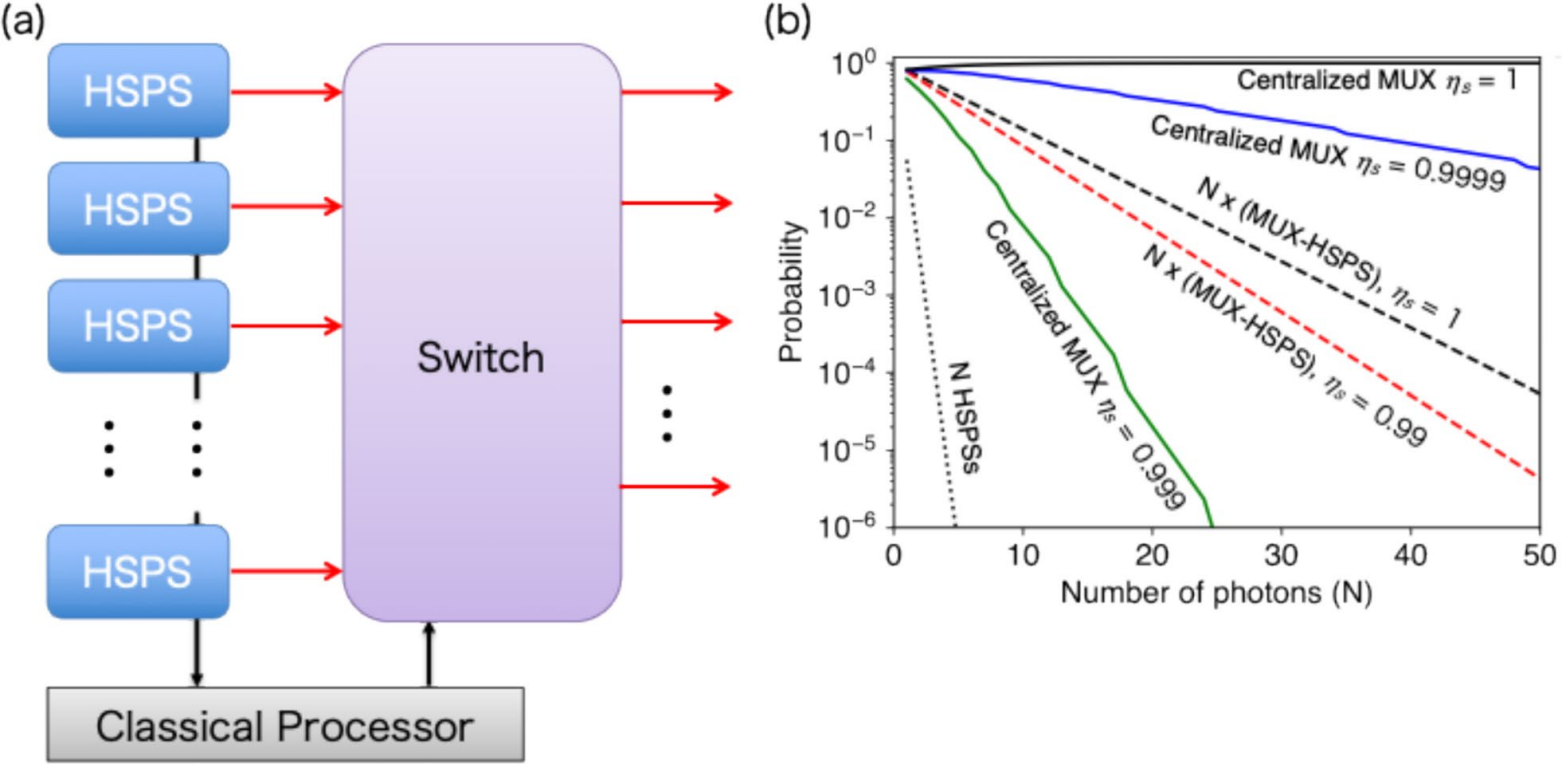
**a** Schematic diagram of centralized MUX HSPSs for multi-photon generation. The switch efficiently routes *N* single photons from $N\times M$ HSPSs. **b** N-photon generation probability for different MUX strategies (*M* = 30, *p* = 0.1) for different values of switch efficiency (*μ*_s_ = 0.99, 0.999, 0.9999, 1). The centralized MUX can outperform *N* sets of *M*-mode MUX-HSPSs, but is highly sensitive to the switch efficiency

For further enhancement of the generation probability, one can consider a centralized MUX of *MN* HSPSs employing a non-blocking $MN\times N$ switch [67, 68, 70], instead of *N* sets of MUX-HSPSs, as shown in Fig. 5a. The scheme is beneficial because *N* photons can be routed whenever *N* or more of the *NM* heralded sources are heralded. However, a critical trade-off emerges due to switch loss. In the case of a centralized S-MUX with a Benes network consisting of $2\times 2$ switches, the switch network depth (i.e., the number of $2\times 2$ switches a single photon traverses from input to output) is calculated as ${2\mathrm{log}}_{2}MN -1$. Consequently, the corresponding transmission of *N* single photons is ${\eta }_{s}^{{2N\mathrm{log}}_{2}(MN)-N}$. While this switch network depth scales logarithmically, it is deeper than *N* independent S-MUX-HSPSs using binary-tree switches, which require ${\mathrm{log}}_{2}M$ switches per photon, resulting in a total transmission of ${\eta }_{s}^{N{\mathrm{log}}_{2}M}$. Furthermore, the total number of switches for the centralized scheme is $MN{\mathrm{log}}_{2}(MN) - MN/2$, which is also larger than the total count for *N* set of S-MUX-HSPSs ($NM-N$). It is worth noting that multi-mode interferometers can be employed to minimize the number of active phase modulators, albeit at the cost of an increased number of beam splitters [70, 71].

Figure 5b shows the probability of *N*-single-photon generation across various S-MUX strategies and varying $2\times 2$ switch transmission ${\eta }_{s}$. We used $M = 30$ per produced photon and ${p}_{1} = 0.1$ for the plot of all cases. $N$ independent MUX-HSPSs ($N$ MUX-HSPSs) demonstrate significantly higher probabilities—several orders of magnitude—compared to *N* HSPSs without MUX. Crucially, the centralized MUX-HSPS outperforms the $N$ MUX-HSPSs for lossless or extremely low-loss cases (${\eta }_{s}=1, 0.9999$). However, for ${\eta }_{s}=0.999$, which is around the value achieved by the current state of the art, the centralized case yields a lower probability than $N$ MUX-HSPSs with ${\eta }_{s}=1$ and even with ${\eta }_{s} = 0.99.$ This clearly illustrates the paramount importance of minimizing switching loss and optimizing MUX strategies. While current technology cannot yet achieve MUX of so many HSPSs (e.g., 1500 sources for $N=50$ and $M=30$), the potential scalability of MUX for multi-photon generation strongly motivates further development. This is particularly critical for all-optical quantum networks [72] and photonic quantum computing, which require a large number of photons, as will be discussed later.

### MUX of single-mode Fock state generation and photon-number manipulation

Another extension of MUX-HSPS is the generation and manipulation of quantum states in the photon-number Fock basis. Fock states are fundamental photonic quantum states that can be applied for the high-efficiency two-photon linear-optic gate operations as well as for the injection of non-Gaussian photon-number distribution to continuous-variable (CV) photonic quantum computing [73]. In HSPS, by introducing a PNRD instead of a threshold detector, general photon-number states can be heralded according to a thermal photon-number statistics of a TMSV state (with a maximum probability of heralding ${p}_{N}=\frac{{N}^{N}}{{(N+1)}^{N+1}}$ for $\mu =N$). Such sources of heralded photon-number states can be multiplexed similarly to that of HSPS. Despite no observation of an enhanced generation rate, an experiment by Bouillard and co-workers successfully stored a two-photon Fock state in an all-optical loop for 325 ns (25 cycles) with > 50% fidelity to a pure two-photon state [74].

**Fig. 6:**
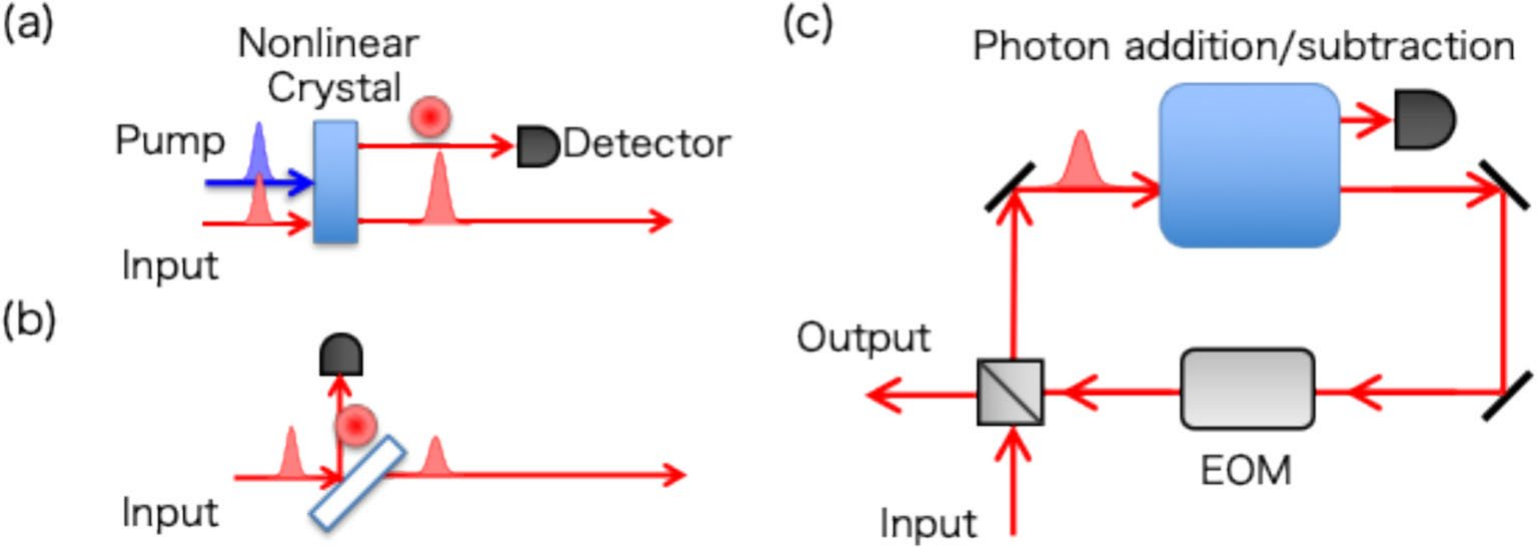
Schematic diagram of PUs for single-mode photon **a** addition and **b** subtraction. **c** T-MUX setup of a photon addition/subtraction PU

A nonlinear source can also be applied to the heralded photon-addition operation [73], as shown in Fig. 6a. Here, an input state is an arbitrary state in the photon-number basis (unlike the vacuum input of HSPS), with the other optical modes matched to the signal mode of a nonlinear source. Since the two-mode squeezing operation produces an identical number of photons on both signal and idler modes, photon addition on the signal mode is heralded when an idler (ancilla) photon is created and detected. Photon subtraction can be implemented by a low-reflectivity beam splitter [75], which reflects off a single photon in an input state, as shown in Fig. 6b. The detection of a reflected single photon heralds the subtraction of one photon from the transmitted state. Photon addition and subtraction are non-Hermitian, nonlinear operations, and are essential for implementing non-Gaussian operations in CV quantum computing and for enhancing the success probability of linear-optic quantum gate operations, as will be discussed in Sect. 4. However, despite their importance in both photon-based and CV quantum information applications, these photon-number manipulations are probabilistic due to the uncertainty of the number of added/subtracted photons. Thus, such an experiment is typically performed with a low gain (i.e., low pump pulse energy and low beamsplitter reflectivity) to ensure a single-photon addition/subtraction, albeit at the cost of a low heralding probability.

The probabilistic photon addition/subtraction can be efficiently overcome by a T-MUX scheme based on the proposal by McCusker and Kwiat [76]. As shown in Fig. 6c, a photon adder/subtracter used as a PU is placed inside the optical loop (as the conceptual diagram of T-MUX in Fig. 1b), and then repeatedly pumped with a period $\tau$ that matches the round-trip time of the loop. When an initial state of the T-MUX system is a vacuum state, the number of photons created in the loop equals the sum of the heralded photon numbers of all preceding pumping events. Photons in the loop are released from the loop when a desired number of photons is heralded. This T-MUX is permissible because if no heralding signal is detected, the input quantum state remains unchanged (assuming negligible loss in the optical system and detectors). Therefore, the T-MUX system can reliably repeat the operation of the low-yield PU to build up a number of photons, and the success probability approaches unity for a sufficient number of cycles *M*. In single-photon addition/subtraction, the scaling of the probability is analogous to Eq. (1) for single-photon generation. The success probability and fidelity in manipulating higher photon numbers with imperfect components are discussed in [76]. In general, the T-MUX scheme is also applicable to the external input state from outside the loop, which can thus generate a non-Gaussian quantum state from a Gaussian one. T-MUX of photon subtraction can be similarly operated by using the probabilistic photon subtraction unit. Note that the heralding probability of these operations depends on the number of photons in the input state. For example, one can have a higher likelihood of single-photon subtraction from a higher-number state. By using additional optical switches, the nonlinear source gain and the beam splitter reflectivity can be adaptively tuned to optimal values to achieve high-precision photon-number manipulation with a minimum number of attempts.

The T-MUX photon-addition setup can be further extended to generate two-mode photon-number entanglement [76], including the N00N state [77]. A N00N state consists of *N* photons, which is present in either of two modes $\frac{1}{\sqrt{2}}\left(|N0\rangle +|0N\rangle \right)$. This type of entanglement has proved useful for quantum-enhanced precision measurement (metrology) beyond the standard quantum limit [78–80]. N00N states for *N* = 2 are directly created by a nonlinear photon pair source [81–83] and have been successfully utilized for quantum precision measurements beyond the standard quantum limit [84]. However, higher photon-number N00N states can be produced by only postselective measurements [85, 86] due to the probabilistic generation of multiple photon pairs and the uncertainty of their produced modes. The T-MUX scheme relies on the fact that the N00N state is decomposed into a product of *N* single photons in dual-rail superposition states [87]. Thus, by rotating the polarization of photons in the loop each time after the photon-addition operation is heralded, the N00N state is quasi-deterministically generated into orthogonal polarization modes. The scheme can also produce more general two-mode photon-number entangled states $\frac{1}{\sqrt{2}}\left(|NN^{\prime}\rangle +|N^{\prime}N\rangle \right)$, which is more robust against loss to achieve quantum-enhanced precision measurements.

## MUX of dual-rail state generation and quantum computing

The realization of general quantum information applications fundamentally requires the generation and manipulation of multi-mode quantum systems featuring both superposition and entanglement. Central to this approach is the KLM dual-rail encoding [18], which has become the standard method for representing a logical qubit as the presence of a single photon in one of two orthogonal modes; $|01\rangle$ and $|10\rangle$. Its popularity stems from its inherent simplicity and tolerance to optical loss. However, MUX of such complex dual-rail multi-photon states and entangled states presents significantly greater technical challenges than MUX of single-mode HSPSs. These challenges are compounded by higher resource requirements, the low success probability of PUs, and the demanding complexity of active feedforward operations required for conditional state preparation. In this section, we review crucial proposals for MUX techniques that aim to achieve high efficiency in the heralded generation of dual-rail entangled states. These schemes are essential building blocks for photonic measurement-based quantum computing (MBQC) architectures, potentially realizable through the rapid integration of advanced hardware components in the near future. We also see that MUX efficiently implements fusion-based quantum computing (FBQC) [88], a promising MBQC architecture. Furthermore, we see that MUX strategies play a similarly vital role in the development of CV-MBQC.

### Dual rail entanglement generation

**Fig. 7:**
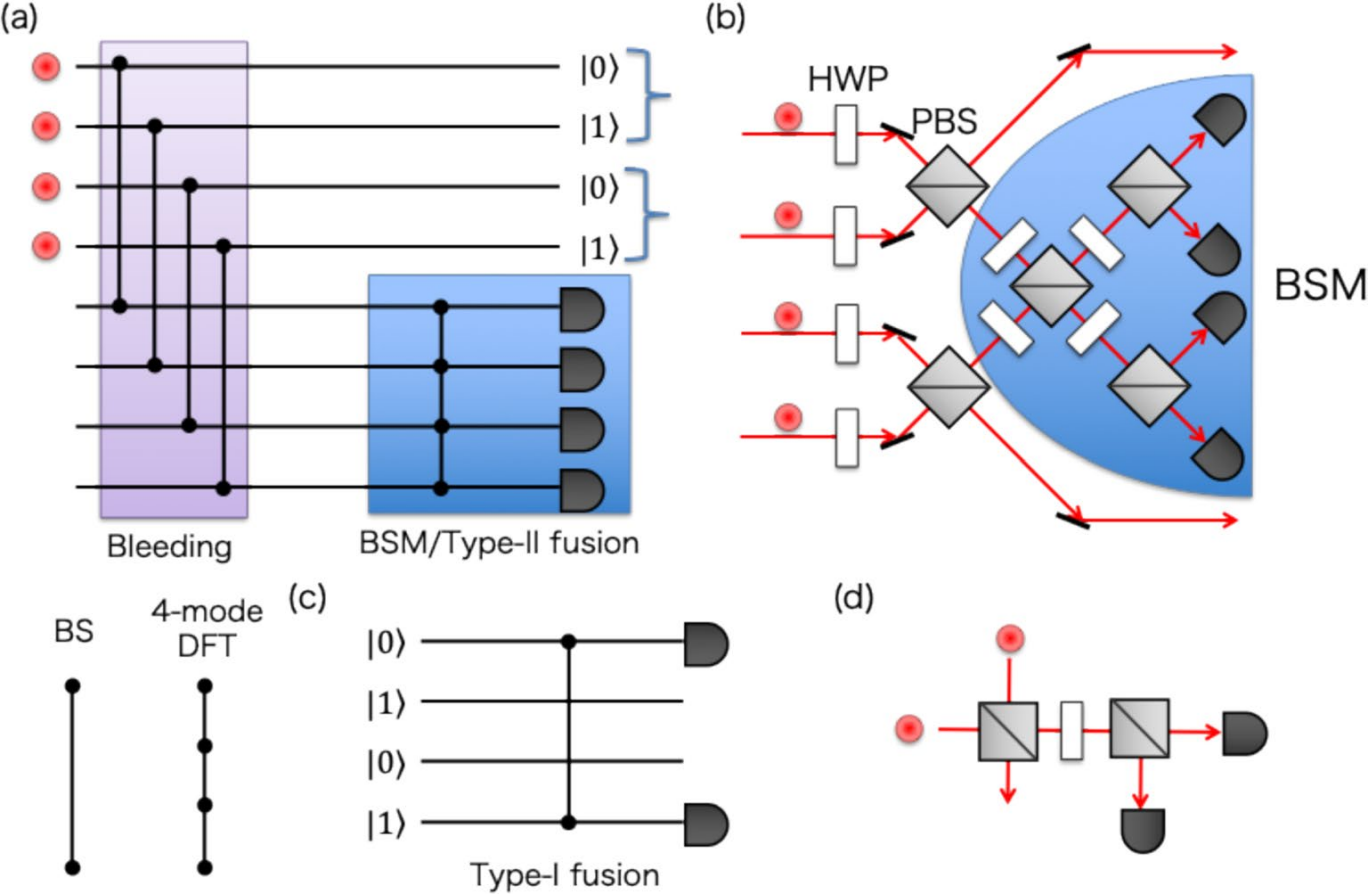
**a** Schematic diagram of a Bell state generator, which consists of tunable beamsplitters and Bell-state measurement (BSM), which can also serve as type-II fusion of two entanglement systems. The scheme is compatible with the T-MUX bleeding technique. **b** Heralded generator of in polarization-encoded Bell states proposed in [91], which is compatible with T-MUX bleeding and boosting operations. **c** Schematic diagram of type-I fusion gate and **d** corresponding experimental setup of **c** in polarization-encoded qubits. BS, beam splitter; DFT, discrete Fourier transform; HWP, half-waveplate; PBS, polarizing beamsplitter

Generation of dual-rail entangled photons is the first step of photonic quantum information applications. Extensions and improvements of schemes have been proposed continuously, particularly in the generation of Bell states, which involves the entanglement of two dual-rail qubits; $|{\phi }^{\pm }\rangle =\frac{1}{\sqrt{2}}\left(|01\rangle |01\rangle \pm |10\rangle |10\rangle \right)$ and $|{\psi }^{\pm }\rangle =\frac{1}{\sqrt{2}}\left(|01\rangle |10\rangle \pm |10\rangle |01\rangle \right)$. In principle, MUX can enhance the probability of any heralded generation schemes [89–95], as demonstrated for HSPSs (various Bell-state generation schemes are reviewed in [96]). A relatively high-efficiency Bell-state generation scheme has been proposed and demonstrated by Zhang and coworkers [91], as shown in Fig. 7a, b: four single-photon states at different optical paths are transformed to a superposition of pairs of four Bell states via 50:50 beamsplitters when two of four photons are reflected with the probability of 3/8. The reflected two photons are then subjected to Bell-state measurement (BSM) [27] in four ancilla modes, where the projection onto a Bell state is achieved by detecting two photons at different detectors. The projection is also probabilistic (< 50%) with linear optics [91], but heralds the transformation of undetected photons into a Bell state (e.g., the setup shown in Fig. 7b performs deterministic projection of input states onto $|{\phi }^{+}\rangle$ and $|{\psi }^{+}\rangle$ but does not onto $|{\phi }^{-}\rangle$ or $|{\psi }^{-}\rangle$). Thus, this scheme contains three probabilistic processes; generating four single photons (${p}_{1}^{4}$), routing two of the four photons to the BSM setup (3/8 for 50:50 beamsplitters), and projecting two photons into a Bell state (1/2) even for a lossless system: The total success probability for the ideal non-MUX case is ${3p}_{1}^{4}/16$.

MUX techniques can address all probabilistic processes in this Bell-state generation scheme: first, MUX-HSPSs discussed in Sect. 3 can simultaneously prepare four single photons quasi-deterministically. Second, the two-photon routing process can be performed deterministically using the bleeding technique, i.e., the T-MUX photon subtraction proposed by the team at PsiQuantum Inc. [97]. By using beamsplitters with a low transmission probability, not 50%, the likelihood of directing more than two photons to the BSM setup can be made arbitrarily small. Although the likelihood of directing two photons is also reduced, photons transmitted through beamsplitters are re-injected into the optical loop architecture. One can repeat this process until the detectors in the BSM setup detect two photons. The sequential two-photon detection can be equivalent to their simultaneous detection case in this particular setup, since a no-photon detection event in BSM heralds an identity operation on the input four-photon quantum state, and the measurement operators for single-photon detection at four detectors commute with each other [97]. Thus, the bleeding operation achieves quasi-deterministic two-photon detection in the BSM setup. Note that this T-MUX bleeding strategy inevitably reduces the operational rate of Bell state generation due to the necessity of repeating low-probability operations. In practice, one must optimize the reflectivity of the beamsplitters (in each cycle) to ensure the process completes within a finite number of cycles, thereby mitigating accumulated optical loss in the loop (which is critical, as it must store up to 4 photons simultaneously), as discussed in [97].

Furthermore, the success probability of the linear-optic BSM can be boosted by utilizing ancilla photons [98, 99], which enables probabilistic projection onto $|{\phi }^{-}\rangle$ and $|{\psi }^{-}\rangle$ in addition to deterministic projection onto $|{\phi }^{+}\rangle$ and $|{\psi }^{+}\rangle$. In general, 2*N* ancilla photons can enhance the success probability of type-II fusion to $1-1/{2}^{N+1}$ [98]. Ancilla photons can be quasi-deterministically produced by MUX-HSPSs. Table 1 presents a summary of the success probability with and without exploiting MUX strategies for generating a Bell state with lossless components, along with related experimental results. The comparison clearly shows the potential of MUX to overcome the probabilistic nature of linear-optical systems in generating the dual-rail entanglement. Performance prediction involving losses is complicated (but possible) due to the uncertainty of the bleeding cycle. However, given the required two-photon generation (for boosting) and storage of more than two photons in each bleeding cycle, a low-loss implementation is essential to achieve enhanced generation. Recent experiments have demonstrated boosted BSM using two ancilla photons in a separable state [100] and a dual-rail entangled state [101] with postselection, achieving the success probability of 57.9% and 69.3%, respectively.

**Table 1:** Comparison of ideal MUX and ideal non-MUX strategies in the Bell-state generation and the necessary operations. Related experimental demonstrations are listed alongside

Operation	Ideal non-MUX	Ideal MUX	Experiment
HSPS	${p}_{1}^{4}$	1	0.67^4^ (T-MUX HSPS [33])
Photon subtraction	3/8	1	~ 3/8 (non-MUX [91])
Type-I, Type-II Fusion	1/2	3/4 (with two ancilla photons)	0.693 (Boosted Type-II, conditional on ancilla-photon preparation [101])
Bell-state generation	$3{p}_{1}^{4}/16$	3/4	$3 \times {10}^{-8}$(non-MUX, QD source [95])

The BSM is used for entanglement swapping [102, 103] and entanglement type-II fusion [104], where two photons in different entangled states are projected onto a Bell state, and then the remaining photons in two entangled states are merged into one entangled state. Thus, boosted fusion of entangled states with quasi-deterministic ancilla-photon input via MUX is essential for scaling the distance of distributed entanglement and the number of entangled photons. Fusion of entanglement can also be performed by a type-I configuration, as shown in Fig. 7c, d. The type-I fusion gate is advantageous in that only one photon detection is necessary for successful operation, at the cost of loss detection being unavailable. Thus, type-I fusion is helpful for growing entangled states from small ones (e.g., Bell states), while type-II fusion is beneficial in the fusion of larger entangled states with loss tolerance via two-photon detection. Boosted type-I fusion is also possible, as is type-II fusion, thereby increasing the success probability from 1/2 to 3/4 by using two ancilla single photons [97].

**Fig. 8:**
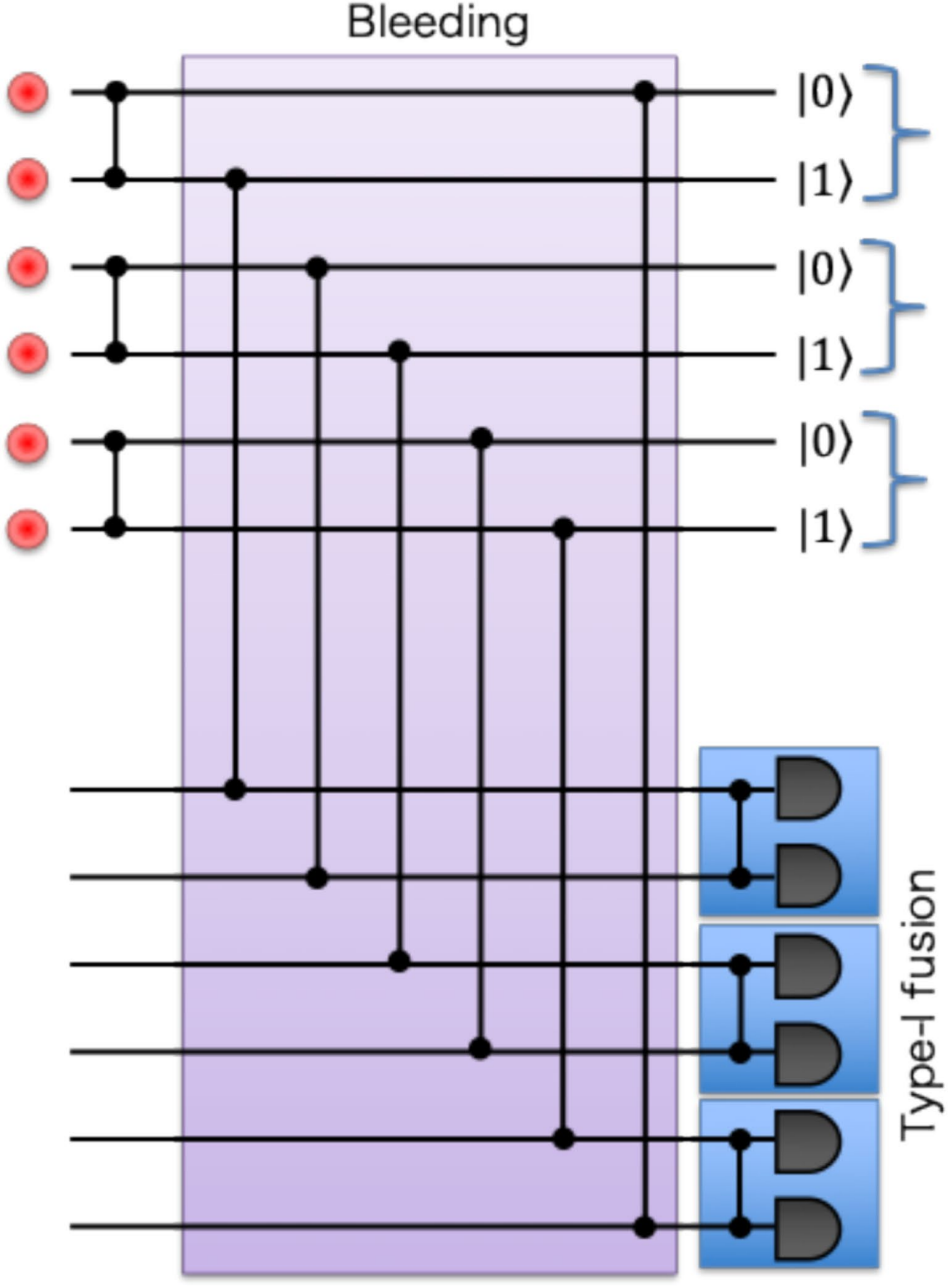
Schematic diagram of 3-photon GHZ state generator. Boosted fusion and T-MUX of bleeding are also applicable to the scheme, similar to the Bell-state generator shown in Fig. 7a

The type-I fusion and the bleeding technique via MUX can also be applied to the generation of Greenberger-Horne-Zeilinger (GHZ) states [105] $$ \left| {{\mathrm{GHZ}}_{N} \rangle } \right. = $$$$ \frac{1}{{\sqrt 2 }}\left( {|01\rangle ^{{ \otimes N}} + |10\rangle ^{{ \otimes N}} } \right) $$, which exhibit stronger nonlocality than Bell states and are an essential resource of MBQC. This scheme was also found by the team of PsiQuantum Inc. [97]. Schematics of generating a 3-photon GHZ state $$|{\mathrm{GHZ}}_{3}\rangle $$ are depicted in Fig. 8. In this scheme, input 6 single photons are transformed into 3 N00N states for $$N=2$$, and then subjected to photon bleeding operations to send at most one photon into each Type-I fusion gate. Since the input state is unchanged when Type-I fusion detects no photons, the state can be recycled to re-input bleeding until all Type-I fusion herald their success of operations: photons in heralded fused modes are released from the bleeding loop and then stored at an additional optical storage loop to synchronize the generation between other photons. Thus, MUX techniques in bleeding and boosted type-I fusion achieve enhancement of the success probability of up to 1/4 compared to non-MUX, single-shot operation case ($${p}_{1}^{6}/32)$$ for generating $$|{\mathrm{GHZ}}_{3}\rangle $$. For general *N*-photon GHZ state generation, the extension of the optical circuit to 2*N* input photons and *N* type-I fusion gates with MUX bleeding operation has been theoretically proven to achieve the probability of 1/2^*N*−1^, whereas the non-MUX strategy is limited by 1/2^2*N*−1^ [97]. Besides this advanced MUX scheme, any proposed heralded schemes [106–111] for GHZ-state generation are compatible with S-MUX and T-MUX generation schemes. Experimental demonstration of heralded 3-photon GHZ states has already been reported in several experiments [55, 112, 113].

### Photonic quantum computing: FBQC

For quantum computing with photons, the lack of high-efficiency nonlinearity is a significant limitation for scalability. The renowned KLM scheme [18] utilizing heralded measurement-based nonlinearity has been proven to be a universal quantum computing scheme. However, its original circuit-based architecture demands an impractically large number of sources and requires low-latency sequential feedforward operations with ultra-low loss (< 1% overall loss reported in [114]).

**Fig. 9:**
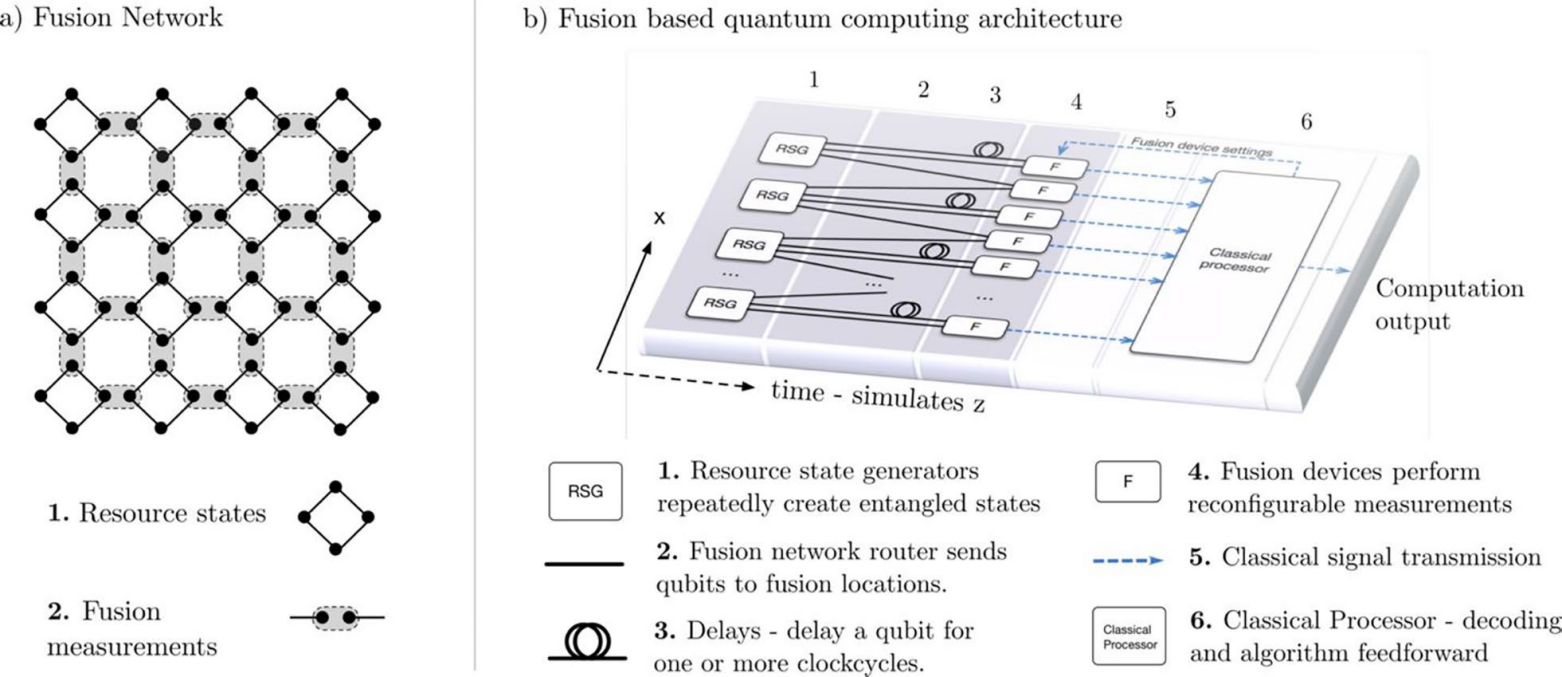
Illustration of fusion-based quantum computing (FBQC) architecture. **a** An example of a 2D fusion network. **b** An example architecture to create the fusion network shown in (**a**). Reproduced under the terms of the CC-BY Creative Commons Attribution 4.0 International License from Ref. [88]

Measurement-based quantum computing (MBQC) was subsequently proposed by Raussendorf and Briegel [115] as a more practical architecture. In this scheme, a large-scale entangled state (a cluster state) is initially created, and then sequential single-qubit measurements coupled with active feedforward operations are performed. Single-qubit measurements of entangled nodes serve as gate operations for the rest of the entangled photons. However, applying MBQC directly to photonic qubits also poses a challenge because of the practical difficulties in the low-loss preparation and storage of a large number of photons required for entangling operations. To overcome this challenge, Browne and Rudolph [104] proposed an MBQC method that repeatedly fuses small-scale entangled states using probabilistic linear-optical gates (akin to KLM techniques). This approach dynamically constructs cluster states, significantly reducing resource overhead while maintaining fault tolerance. The scheme is currently called fusion-based quantum computing (FBQC), which is considered one of the most promising avenues for scaling up photonic quantum computing, and is highly reliant on MUX strategies [88]. The architecture of the scheme has been continuously improved and refined [43, 51, 71, 97, 106, 116]. The architecture of FBQC consists of resource-state generators, optical buffers, switchable (type-II) fusion gates, and a classical processor for active feedforward operations, as shown in Fig. 9 (reproduced under the terms of the CC-BY Creative Commons Attribution 4.0 International License from Ref [88]). Resource-state generators are fully implemented using MUX to generate small-scale entangled photons in cluster states by fusing further small Bell and GHZ states, in a periodic, deterministic manner. Produced photons are sent to fusion gates, where small-scale entangled photons are united into a single state with increased size. The combinations of photons subjected to fusion are predetermined so that the resulting state constructs a network suitable for a topological error-correcting code. Measurement results in fusion gates are analyzed, and active feedforward is applied to subsequent fusion and single-qubit measurements.

Fusion operations are performed on resource states produced in parallel (simultaneously) and those produced sequentially: most of the photons in a resource state are subjected to fusion with those in other resource states produced simultaneously, and some photons pass through a delay line to be subjected to fusion with photons in subsequently produced resource states. This S-T-MUX of fusions enables the construction of a large-scale entanglement. Most of the photons are subjected to fusion — a destructive measurement — within their finite lifetime, meaning that all photons constructing a large-scale entanglement do not necessarily have to exist simultaneously, thanks to the entanglement in each resource state and the space–time fusion networks.

The MUX of fusions plays a vital role in the construction of entanglement and in error detection and correction. A successful type-II fusion consumes two input photons with the measurement outcomes of two joint Pauli operators (e.g., $\widehat{{Z}_{i}}\widehat{{Z}_{j}}$ and $\widehat{{X}_{i}}\widehat{{X}_{j}}$), an action known to propagate entanglement from the measured qubits to their entangled neighbors. The fusion is probabilistic and can fail as an unsuccessful projection onto a definite entangled state, corresponding to the single-qubit measurements of two single photons (e.g., $\widehat{{Z}_{i}}\widehat{{Z}_{j}}$). This unsuccessful projection does not contribute to entanglement scaling, but the resource state’s robust, fault-tolerant structure allows the system to discard the failed attempt and continue entanglement scaling through successful parallel and sequential fusion operations. In practice, non-heralding erasure events by the detection of fewer than 2 photons (due to photon loss in optical components, switches, or detectors) can also occur. The fusion networks achieve tolerance to some degree of loss by constructing topological codes [88].

Therefore, FBQC is designed under two strict technical constraints: first, the fusion success probability (which can be enhanced by MUX as discussed above) must exceed a threshold that prevents the pipeline from being interrupted for a given time threshold. Second, the photon loss rates of all optical components must be kept below the fault-tolerance threshold of the quantum error correction code. In general, there is a complex trade-off: increasing the success probability of fusion often requires more complicated setups and operations (e.g., bleeding and boosting), which can degrade the overall loss per photon. The loss of current state-of-the-art photonic devices (reviewed in Sect. 5) remains above the tolerance threshold (1–2% loss per photon) [88]. A recent study by Song and coworkers has demonstrated encoded fusion of multi-photon logical qubits, showing superior fault tolerance of over 10% loss per photon [117]. Optimizing the MUX sources and entanglement generation will play a vital role in achieving fault tolerance in practical devices.

### MUX in other photonic quantum computing architectures

While FBQC utilizes S-T-MUX fusion networks, frequency-domain quantum computing has also been proposed as an alternative approach [118–120]. In frequency-bin encoding, single-photon pulses encoded into multiple frequency modes are defined as logical qubits, enabling the simultaneous handling of many independent qubits within a single waveguide or fiber. This method dramatically reduces the complexity of optical paths, offering an inherently low-loss platform and making it highly advantageous for photonic integrated circuit integration. Although a fault-tolerant architecture for this platform may not yet have been proposed, the generation and manipulation of photons in the frequency domain are highly compatible with MUX, which can be used to enhance the effective probability of probabilistic operations, as discussed in the preceding sections.

CV quantum computing can also obtain benefits from MUX techniques. The Gottesman-Kitaev-Preskill (GKP) code [121] is promising for fault-tolerant universal quantum computing. The generation of a high-fidelity GKP state is an inherently probabilistic process, often involving the interference of squeezed light and subsequent photon (non-Gaussian state) subtraction [122, 123] (akin to KLM gates and Gaussian boson sampling [124–127]). The successful generation is heralded by the detection of specific photon-number states and thus can be enhanced by MUX techniques. The research group in Xanadu Quantum Technologies Inc. has demonstrated S-MUX of heralded GKP sources [128] as well as space–time cluster state entanglement, mirroring how MUX is used for photon-based resource state generation and the space–time fusion networks.

Crucially, T-MUX techniques play a foundational role not only in GKP state generation, but also in the architecture of CV-MBQC itself. Techniques for generating large-scale cluster states using T-MUX and for generating a large-scale cluster state [129, 130] and a loop-based quantum computing architecture [131, 132] have been demonstrated using Gaussian states. In these architectures, a single or a few squeezed light sources are used to generate a train of pulses, which are then entangled into a high-dimensional cluster state via delay loops and beam splitters. This approach, combined with the integration of deterministic GKP qubits via MUX, offers a scalable pathway to fault-tolerant CV quantum computation, paralleling the FBQC strategy in the phonon-based (discrete-variable) domain.

We also note that MUX techniques, particularly T-MUX, can be used to implement resource-efficient postselective entangled-photon generation [53, 54, 133]. Boson sampling circuits, which do not need nonlinearity in state manipulation but require large numbers of modes and photons, can be efficiently implemented by the loop-based architecture [126, 134]. Thus, MUX techniques themselves are promising tools for scaling up quantum applications, and their hardware and architectural developments are contributing significantly to various photonic quantum applications.

## Hardware developments

The successful implementation and scalability of MUX, as discussed throughout the preceding sections, critically depend on the performance of its underlying quantum optical components—sources, detectors, switches, and their networks. In this section, we explore the recent hardware advancements to fully assess the potential of MUX techniques for realizing large-scale quantum applications in the near future. Basic operational principles of single-photon sources and detectors are nicely reviewed in [135]. Tables 2, 3, and 4 present comparisons of representative sources, detectors, and switches, respectively, that are demonstrated in or applicable to MUX experiments.

**Table 2:** Comparison of representative photon sources

Type	SPDC (Bulk) [31]	SPDC (Waveguide) [157, 158]	SFWM (Si-PIC) [51]	Quantum Dot (InAs) [57]
Output State	TMSV/HSPS	TMSV/HSPS	TMSV/HSPS	Single Photon
	(Probabilistic)	(Probabilistic)	(Probabilistic)	(Deterministic)
Loss	0.37 dB	3.2–5.2 dB	> 1 dB	1.4 dB
Brightness	3.9 × 10^3^ pairs/mW	3.5 − 5.6 × 10^6^ cps/mW	~ 10^6^ cps/mW^2^*	1.8 × 107 cps**
Source-to-source (Single-source) indistinguishability	0.986	– (0.945)	0.995	–***(0.986)
Wavelength	1550 nm	1540 nm	1550 nm	884 nm
Bandwidth	~ 500 GHz	~ 1 THz	~ 6 GHz	2.1 GHz
Temperature	300 K	300 K	2.2 K (to 300 K)****	< 10 K
Advantages	Low loss	High efficiency	Scalability	Deterministic generation
Challenges	Scalability, Multi-photon generation	Loss, Multi-photon generation	Loss, Multi-photon generation	Inhomogeneity

* Estimated from similar Si-PIC ring resonator sources [176]

** The value is limited by detector count rates

*** 69% source-to-source indistinguishability has been reported in [61]

**** The experiment was performed at 2.2 K due to the integrated SNSPDs, but the source itself may not require cryogenic cooling

**Table 3:** Comparison of SNSPD and TES operating at ~ 1550 nm. For SNSPDs, since (engineering) trade-offs and optimization required for a specific metric often prevent a single device from achieving peak performance across all categories, we show figures of merit of the state-of-the-art SNSPD exhibiting approximately 90% system detection efficiency or higher, along with PNR capability, low timing jitter, and/or high count rates

Type	SNSPD (NbTiN) [188])	SNSPD (NbN) [189]	SNSPD (NbN, 32 pixels) [195]	SNSPD (NbTiN, 28 pixels) [191]	TES (W) [128]
System detection efficiency	99.5%@1350 nm 94%@1500 nm	97.4%@1550 nm, 98.2%@1590 nm	97.5%@1555 nm	88%@1550 nm	97%
Dark count rate	300–500 cps	< 100 cps	20 cps	60 cps	< 0.1 cps*
Timing jitter	30 ps	66 ps	382 ps (for 1 photon), 40.4 ps (for 32 photons)	< 80 ps	58 ns*
Maximum count rate	~ 10 Mcps**	20 Mcps**	41.7 Mcps**	~ 250 Mcps** (> 1 Gcps with 4 chips)	> 1 MHz
PNR capability	No	No	Pseudo-PNR (8 photons)	Pseudo-PNR (8 photons)	True-PNR (7 photons)
Operating temperature	2.7 K	0.8 K	0.85 K	0.8 K	0.012 K

* Values inferred from [218] and [211]

** Count rate at -3 dB efficiency

**Table 4:** Comparison of photonic switches

Type	EOM (Bulk RTP) [33]	EOM (TFLN Waveguide) [235]	EOM (Waveguide BTO) [51, 236]	XPM (Fiber) [174]	BSFWM (Fiber) [48]
Insertion Loss	0.03 dB@1550–1590 nm	0.4 dB@730 nm (On chip)	0.2 dB@1550 nm (On chip)	0.8 dB@1550 nm	1.3 dB@1300 nm (internal conversion loss of 0.32 dB)
Rise Time	3 ns	~ 10 ps	~ 20 ps	200 ps*	2.5 ns
Operational Rate	1 MHz	40 GHz	20 GHz	200 MHz	1 MHz
Noise Photon Rate	Negligible	Negligible	Negligible	$4\times {10}^{-6}$/pulse	$3\times {10}^{-3}$/pulse
Advantages	Low loss	High scalability & Speed	High scalability & Speed	High speed	Multi-mode frequency conversion
Challenges	Low speed	Loss	Loss	Loss, Noise	Loss, Noise, Efficiency

* A 1.7 ps rise time has been demonstrated at 800 nm in [170]

### Basic single photon sources

As discussed in Sect. 3.1, basic photon sources, such as probabilistic HSPS and deterministic quantum dot single-photon sources, have been widely used in quantum optics experiments. The quality of the sources ultimately determines the fidelity of gate operations and the scalability of a MUX system. Photons need to be produced with high brightness, low loss, and high indistinguishability, which are associated with system efficiency, photon-number fidelity, and quality of two-photon interference [136] for quantum gate operations.

HSPSs implemented by bulk-optic SPDC have achieved source-to-source indistinguishability (> 95%) and low-loss photon collection into single-mode optical fibers (< 5%) [30, 31, 84]. Engineered group-velocity-matching (GVM) conditions [30, 137–142] and modulated poling structures [31, 143–148] enable the direct generation of heralded single photons without spectral filters, which typically add loss [149]. Low-loss photon collection into single-mode optical fibers has been achieved by utilizing spatial entanglement of photon pairs [30, 31, 84, 150–154]. Bulk optics HSPSs are not well compatible with large-scale S-MUX due to their large footprints, whereas T-MUX can be efficiently implemented with low-loss bulk optics components [33, 34, 63]. Waveguide-type nonlinear sources improve the low generation efficiency because the involved optical modes are confined into a small area and thereby interact more strongly than their bulk-optics counterparts. Although structure-oriented (waveguide) dispersion should be taken into account to achieve a GVM condition, a ~ 1000-fold enhancement of the generation rate per pump power with high indistinguishability has been demonstrated [155–158]. The improvement of waveguide propagation loss (0.07 dB/cm) and waveguide-to-fiber coupling loss (3%) has also been reported [159, 160]. Thin-film lithium niobate (TFLN) on insulator has recently emerged as an integration platform, enabling monolithic integration of SPDC sources [161] and ultra-fast EOM [162]. A typical pair-generation probability for both bulk and waveguide SPDC sources is < 5%, much lower than the 25% limit of HSPS, to mitigate the likelihood of incorrect heralding of multi-photon states with threshold detectors. However, high-efficiency PNRD allows the use of a higher mean photon number and generation probability while maintaining high photon-number fidelity, as discussed in the following subsection.

HSPSs based on the SFWM process have also been realized by various platforms with two-dimensional confinement structures. HSPSs utilizing photonic crystal fibers [163, 164], telecommunication optical fibers [165–167], and laser-written waveguides in glass [168, 169] have been demonstrated at around their zero-dispersion wavelengths. However, the glass-based SFWM sources typically accompany noisy Raman scattering, necessitating that the nonlinear medium be sufficiently cooled down, and/or photon pairs be produced outside the Raman gain spectral range (~ 40 THz) [165]. Nonetheless, in-fiber photon-pair and heralded single-photon generation are beneficial for lossless coupling to optical fibers (although pump pulses must be eliminated after the SFWM process). Furthermore, the third-order nonlinearity inherent to the fiber platform is also useful for all-optical switching [170–174] and quantum information processing in T-MUX and F-MUX.

SFWM sources in silicon and silicon nitride (SiN) photonic integration circuits (PICs) have been advancing rapidly due to their promising compatibility and scalability via S-AMUX [51, 175, 176]. Although chip-to-fiber coupling efficiency requires further improvement for out-of-chip quantum applications, when photons do not have to be extracted from a chip (such as in on-chip quantum computing applications), high indistinguishability (> 99%) utilizing group delays between different waveguide modes [177] and cascaded resonator configuration [51] has been achieved at a telecommunication band.

As discussed in Sect. 3, quantum-dot-based single-photon sources can produce single photons deterministically. Although source-to-source indistinguishability remains an ongoing challenge [61], multi-photon states can be prepared by the parallel-to-serial conversion. Beyond these established platforms, emerging material systems and nanostructures are paving the way for fully integrated sources essential for realizing large-scale S-MUX. 2D materials, such as hexagonal boron nitride (hBN) and transition-metal dichalcogenides (TMDCs, e.g., WSe_2_) [178], are rapidly gaining attention as promising candidates for on-chip single-photon emitters. Unlike traditional epitaxial quantum dots, these 2D materials offer superior compatibility with monolithic integration onto SiN waveguides due to their atomic-layer thickness and ease of transfer [179–181]. Similarly, color centers in silicon carbide (SiC) are emerging as a robust, room-temperature single-photon source that combines with mature wafer-scale fabrication [182]. Table 2 shows the figures of merit for the representative photon sources targeting the ~ 1550 nm. Note that the high-performance quantum dot source listed operates at 884 nm [60], reflecting the current state-of-the-art for that platform.

### Photon detectors

Photon detectors trigger MUX operations, and thus, their detection efficiency, timing jitter, and maximum count rate directly affect the operational precision and speed of MUX. In addition, recent progress in experimental techniques and architecture, such as MUX, has highlighted the importance of PNRD. The photon-number fidelity of HSPSs is largely affected by detector efficiency and photon-number-resolving capability.

Semiconductor (e.g., Si, InGaAs) avalanche photodiodes are traditionally used as threshold single-photon detectors, operated at room temperature, with moderate efficiency (~ 30–70%) at the visible to near-infrared wavelength range, timing resolution (~ 10–100 ps), and dark count rate (< 100 cps) [135, 183]. A critical drawback of semiconductor single-photon detectors is their low efficiency (~ 30%) in the telecommunication band, where other quantum optical devices and optical fibers can operate at low loss. This type of detector is useful for single- and few-photon applications implemented outside laboratories, such as quantum LiDAR [184].

Superconducting nanowire single-photon detectors (SNSPD), which were first demonstrated in 2001 by Gol’tsman and coworkers [185], are a nanowire (typically meandering with a wire width of < 100 nm) made of a thin film of superconducting material (e.g., NbN, WSi, NbTiN, MoSi). An SNSPD is operated with a bias current slightly below its critical current. When the nanowire absorbs a single photon, it causes a local break of superconductivity, producing a short electric pulse as a signal of single-photon detection. SNSPDs require placement in a cryogenic system, typically operated at ~ 0.1–2.5 K, much lower than their transition temperature to mitigate thermal transition and dark counts. Since the groundbreaking work by Nam’s group in NIST [186] demonstrating 93% system detection efficiency, < 1cps detector dark count rate, 150 ps timing jitter, and > 20 MHz count rate simultaneously at the telecom band (1550 nm), SNSPDs have been continuously improved and have become the current standard detectors for quantum optics experiments across a wide wavelength range (0.4–10 microns). The current state of the art demonstrates > 98% system detection efficiency [187–189], < 5 ps timing jitter [190], and > 1 GHz count rate [191–194] at 1550 nm.

Moreover, PNRD utilizing SNSPDs has been reported in recent studies. A straightforward method is to implement detector trees or arrays consisting of multiple threshold detectors. When the number of photons is sufficiently small compared to the number of detectors in a tree/array, the detector system functions as a pseudo-PNRD; incorrect photon counting (undercounting) occurs when multiple photons end up at a single detector. Recent experiments have demonstrated ~ 30 detectors [191, 195] and 100 detectors [196] implementing the pseudo-PNRD. True PNRD with a single SNSPD has also been demonstrated by utilizing the photon-number dependency of the response time (rising edge) [197–200], although the crosstalk between signals of adjacent photon numbers (*N*- and (*N* + 1)*-*photon states) is increased as *N*, limiting the PNRD operation for up to *N* = 7 [199].

Fabricating the nanoscale structures of SNSPDs requires advanced nanofabrication techniques such as electron beam lithography (EBL), which poses challenges for reproducible, efficient fabrication. Recent demonstrations of superconducting microstrip single-photon detectors (SMSPD) [201–205] and superconducting widestrip single-photon detectors (SWSPD) [206, 207] overcome the difficulty of the nanofabrication problems. SMSPDs fabricated with ~ 1 μm strip width have already demonstrated > 90% detection efficiency [208] and 10-photon-number resolution [209]. SWSPDs have a > 10 µm strip width, enabled by introducing a high-critical-current bank structure. This structure suppresses the intrinsic dark counts and enables sufficient biasing of superconducting current across the wide strip. Consequently, high-efficiency photon detection is achieved using wide strips that can be fabricated with optical lithography, which is less costly and has lower resolution than EBL. SWSPDs have demonstrated polarization-independent operation, enabling operation without polarization controllers to improve system stability. Such high-performance detectors with reduced fabrication precision and complexity will be crucial for advanced MUX operations. Recent progress and details of SNSPD devices are reviewed in [210–212].

Transition edge sensors (TES) are another type of superconducting detector that serves as true PNRD devices with high detection efficiency (> 95%) [213–216]. TES serves as a highly sensitive thermal (energy) sensor using a superconducting thin film that operates at cryogenic temperatures (~ < 100 mK), slightly below its critical temperature. The sensor film is biased at a constant voltage at its superconducting transition edge, where the resistance is significantly changed upon absorption of light, whose energy depends on the number of photons and frequency. Thanks to the true PNRD feature, a non-classical (sub-Poissonian) photon-number statistics has been demonstrated in the detection of heralded photon-number states for up to 50 photons [217]. Despite its typical large timing jitter (10–100 ns) and low operational rate (~ 100 kHz), TESs with an improved operational rate (1 MHz) [218] have successfully demonstrated S-MUX of subtracting multiple photons to generate GKP states [128], in which subtraction of a larger number of photons is more desirable to achieve stronger non-Gaussianity. The figures of merit for SNSPDs and TES operated at ~ 1550 nm are listed in Table 3. For SNSPDs, since (engineering) trade-offs often prevent a single device from achieving peak performance across all categories, we present several studies that exhibit approximately 90% system detection efficiency or higher, along with high count rates, low timing jitter, and/or pseudo PNR capability.

### Optical switches

Optical switches are the drive mechanism of MUX technologies. Many switches compatible with single photons have been developed to realize MUX experiments. Switches incorporated with delay lines and classical processors must adaptively change quantum states in response to detection signals with low loss, high precision, and fast transition times. Since MUX requires real-time, high-speed feedforward control in the nanosecond regime or faster, slow switches, such as thermo-optic switches, are fundamentally unsuitable, despite their benefits in static, reconfigurable circuits [21].

The most common and high-speed switching technology for MUX is the phase modulation by EOMs. The method typically utilizes the Pockels (first-order EO) effect in ferroelectric properties of LN and rubidium titanyl phosphate (RTP), where an applied voltage linearly modulates the refractive index and birefringence. Bulk EOMs [33, 34, 36, 63, 219] exhibit a fast transition time (~ 1–10 ns) and very low insertion loss (< 1%) by applying ~ 1 kV for π phase shift. Waveguide EOMs [39] can achieve faster transition time (< 100 ps) but often suffer from significantly higher loss (typically > 3 dB). This high loss is primarily due to the challenging chip-to-free-space or -fiber coupling interfaces, highlighting a critical trade-off between switching speed and total system loss in integrated versus bulk implementations. Optical ceramic switches (e.g., lead lanthanum zirconium titanate) can also be electrically controlled with a transition time of 10–100 ns and ~ 1 dB loss [38, 40, 65, 220].

All-optical nonlinear cross-phase modulation (XPM) is another major phase-modulation scheme that can surpass EOMs in switching transition time (~ 1–100 ps) [170, 173, 174]. Optical fiber is an optimal platform for XPM, providing sufficient interaction length for π phase shift and near-lossless coupling to another fiber cable for propagation. However, system-level losses (e.g., from pump add/drop ports) and Raman scattering noise must be carefully mitigated.

By incorporating the phase modulators discussed above and an interferometer (e.g., a Mach–Zehnder Interferometer, MZI), spatial switching of optical paths can be implemented. In general, a spatial switch must route photons while strictly preserving their quantum state of all other degrees of freedom. However, this requirement is often not satisfied by standard MZIs or EOMs, which typically exhibit polarization-dependent transmission and phase shifts. This limitation is particularly problematic for free-space experiments, where polarization is the natural choice for dual-rail qubit encoding. Bulk-optic low-loss polarization-maintaining spatial switching has been demonstrated by polarization-maintaining optics with a near-normal angle of incidence and two cross-aligned EO crystals with opposite applied voltages [221, 222]. The switch has been successfully used to route an arbitrarily polarized single photon and a polarization-mode two-photon entanglement N00N state with 1.3% loss, illustrating the capability to switch the spatial and temporal modes (with incorporated into an optical loop) of polarization-encoded qubits. When polarization encoding is not used, polarization-dependent phase modulation (i.e., polarization rotation) and a polarizing beamsplitter serve as a stable spatial switch without relying on a multi-path interferometer. In PICs, the polarization dependency of the EOM is often less critical because waveguides are highly polarization dependent, and qubits are typically encoded in two waveguides.

These spatial switching techniques are directly applicable to time-domain switching, serving as the core mechanism for an all-optical, loop-based quantum memory [33, 34, 63, 126, 131, 158, 223–225], which exhibits low loss (~ 1%) and high bandwidth (> 1 THz). The optical loop can be implemented using high-reflectivity mirrors in free space or an optical fiber cable. In most cases, the overall storage loss is determined by the loss incurred in the high-speed optical switch. Thus, the improvement of spatial switching devices, or ultimately, the loss of phase modulators, will directly contribute to the improvement of time domain switching devices. In principle, optical quantum memories [226] (e.g., rare-earth-ion-doped crystals, diamond color centers, and hot atomic vapor) based on photon absorption and emission in a matter system also work as time-domain switches. Matter-based memories offer long storage times and high storage fidelity, but their narrower bandwidth (~ < 100 GHz) and higher insertion loss (> 10%) require improvement for integration into high-repetition-rate, feedforward-driven T-MUX schemes.

Beyond spatial and temporal routing, frequency switching can be performed by using phase modulators with high modulation frequency and depth [227, 228]. Frequency-mode switching has also been achieved via nonlinear optical frequency conversion, such as difference-frequency generation (DFG) [229] and BSFWM [48, 230–232], driven by strong classical laser pulses in engineered nonlinear media (e.g., periodically-poled LN, optical fiber, and Si microring resonator). For quantum applications, the primary challenges are achieving near-unity conversion efficiency required for high fidelity while strictly suppressing noise photons generated by the pump lasers (e.g., SFWM and Raman scattering). The figures of merit for the representative switches introduced above are shown in Table 4.

As seen in Sects. 3.2 and 4.2 in multi-photon generation and FBQC, optimizing the switching network is vital to unlock the full potential of MUX technologies. Since switches are the most critical components to loss, it is essential to construct a necessary high-performance switching network with a minimal number of switching elements and depth. Moreover, theoretical studies in [71] showed that blocking switches and non-MUX (ballistic) approaches utilizing only passive beam splitter networks can achieve high operational probability with a much larger number of HSPSs than MUX cases. The schemes fully implemented by MUX are clearly dominant with the availability of low-loss non-blocking switches. However, when the number of available HSPSs and detectors is significantly increased, hybridization or transition to a ballistic approach becomes a critical option.

### Integration

The ultimate scalability of MUX requires integrating all necessary components onto a single, stable platform. PICs offer the necessary compactness, stability, and manufacturability for realizing the large-mode switch matrices required by scalable MUX architectures. The primary PIC platforms being explored for quantum photonics are Si [51], SiN [51, 128], and TFLN [233, 234]. Si offers high integration density, CMOS compatibility, and a higher third-order nonlinearity, which is beneficial for on-chip SFWM source generation. SiN is favored for its extremely low propagation loss (approaching 0.1 dB/m) and high-power handling capability (essential for SFWM pump lasers), making it ideal for the passive components (e.g., interferometers and delay lines) that dominate the MUX architecture. TFLN is advanced in the availability of 2nd-order nonlinear optical susceptibilities for implementing fast EOMs [235] and bright HSPSs. The integration of HSPSs, EOMs, and SNSPDs onto a SiN chip has been demonstrated by the team of Psi Quantum Inc. [51]. The team also demonstrated the on-chip integration of high-quality barium titanate (BTO) EOM [236], which exhibited an electro-optic coefficient > 1000 pm/V (compared with ~ 30 pm/V for LN). These steps minimize the highly lossy fiber-to-chip coupling interface for the critical heralding signal, which is essential for maximizing the overall system success probability. The figures of merit for the BTO and TFLN EOMs are shown in Table 4.

In addition to integrating components, a chip-scale MUX system must consider photon buffering for feedforward operations, which require time to detect ancilla photons, analyze detector signals, and activate optical switches. Given the ~ 1–10 ns latency of current fast electronics such as application-specific integrated circuit (ASIC) and field-programmable gate array (FPGA), a straightforward solution is chip-to-fiber coupling to delay photons for the preparation of feedforward operations [51, 128]. Proof-of-concept demonstrations have also been achieved for on-chip quantum memories based on an optical loop [237] and atomic frequency combs [238]. Crucially, the total feedforward latency—encompassing the signal processing time and the physical response of the hardware—determines the required buffer time and the maximum operational rate. Even if electronics latency is minimized through advanced integration, the fundamental lower bound on system latency is ultimately constrained by the photon-detection latency, which is estimated to be on the order of 10 ps for SNSPDs [239]. Success in hybrid integration is a crucial step toward ultra-low latency, truly scalable, near-deterministic MUX photonic systems.

## Conclusions

Despite their excellent stability, photons face challenges due to probabilistic operations caused by weak nonlinearity, which restricts the scalability of quantum information experiments, especially in quantum computing. MUX strategies are among the most promising approaches to overcoming the probabilistic constraints and to evolving photonic quantum technology into a scalable system. Through techniques such as heralding and adaptive optical switching, MUX fundamentally increases the success probability of probabilistic operations toward near unity, enabling deterministic quantum state generation and manipulation. The research and development of optical architectures based on MUX principles will continue to be a critical direction for surpassing the scalability barrier in photonic quantum information technology and advancing to the next stage of large-scale quantum applications.

## Data Availability

Data sharing is not applicable to this article as no new data were created or analyzed in this study.

## Abbreviations

AOM: Acousto-optic modulator
ASIC: Application-specific integrated circuit
BSFWM: Bragg-scattering four-wave mixing
BSM: Bell-state measurement
BTO: Barium titanate
CMOS: Complementary metal–oxide–semiconductor
CV: Continuous-variable
DFG: Difference-frequency generation
EBL: Electron beam lithography
EOM: Electro-optic modulator
FBQC: Fusion-based quantum computing
FPGA: Field-programmable gate array
F-MUX: Frequency multiplexing
GHZ: Greenberger-Horne-Zeilinger
GKP: Gottesman-Kitaev-Preskill
GVM: Group-Velocity Matching
hBN: Hexagonal boron nitride
HSPS: Heralded single-photon Source
KLM: Knill-Laflamme-Milburn
LN: Lithium niobate
MBQC: Measurement-based quantum computing
MDI-QKD: Measurement-device-independent quantum key distribution
MUX: Multiplexing/Multiplexed
MZI: Mach-zehnder interferometer
OAM: Orbital angular momentum
PBS: Polarizing beamsplitter
PIC: Photonic integrated circuit
PNRD: Photon-number-resolving detector
PU: Probabilistic unit
QED: Quantum electrodynamics
R-T-MUX: Relative temporal multiplexing
RTP: Rubidium titanyl phosphate
S-MUX: Spatial multiplexing
SFWM: Spontaneous four-wave mixing
SiN: Silicon nitride
SMSPD: Superconducting microstrip single-photon detector
SNSPD: Superconducting nanowire single-photon detector
SPDC: Spontaneous parametric downconversion
SWSPD: Superconducting widestrip single-photon detector
T-MUX: Temporal multiplexing
TES: Transition edge sensor
TFLN: Thin-film lithium niobate
TMDC: Transition-metal dichalcogenide
TMSV: Two-mode squeezed vacuum
XPM: Cross-phase modulation
